# Probing instructed but unnecessary switches of attentional strategy

**DOI:** 10.1007/s00426-025-02147-8

**Published:** 2025-07-08

**Authors:** Svantje T. Kähler, Mike Wendt, Aquiles Luna-Rodriguez, Thomas Jacobsen

**Affiliations:** 1 Experimental Psychology Unit, Faculty of Humanities and Social Sciences, Helmut Schmidt University/University of the Federal Armed Forces Hamburg, Holstenhofweg 85, 22043 Hamburg, Germany; 2 Department of Psychology, MSH Medical School Hamburg, Hamburg, Germany; 3 Institute for Cognitive and Affective Neuroscience, MSH Medical School Hamburg, Hamburg, Germany

## Abstract

It is widely assumed that attentional strategies can be intentionally shifted. Experimental evidence of such adjustment stems almost exclusively from situations associated with changes concerning perceptual stimulus features, stimulus-related contingencies, or response demands, however. In a series of experiments, we investigated intention- (i.e., instruction-) based shifts of attentional strategy in the absence of additional changes in the task/stimulus environment compared with conditions associated with maintenance of the attentional strategy (i.e., keeping task stimuli, responses, stimulus–response assignments, and presentation contingencies constant for conditions of shift and maintenance). Our method involved a probe task procedure diagnostic of the attentional strategy applied (i.e., strong or weak focusing of visual attention on the centrally presented stimulus element). In Experiment 1, participants were instructed to change the strategy after the first half of the trials. Probe task results provided evidence for adherence to instruction. In Experiments 2 and 3, which involved presenting instructional cues on a trial-by-trial basis, adjustment of attentional strategy appeared confined to a high degree of motivation. Experiment 4 suggests the carryover of instructed attentional strategies to a following (probe task) trial when no novel instruction was presented. Our study demonstrates instruction-based shifts in attentional strategy that are discernably unnecessary for solving the current task and occur without support from a change in the task/stimulus environment.

## Introduction

It is widely assumed that perceived stimulus features are represented with varying strength or “activation” depending on the configuration of the cognitive system, often referred to as the *attentional set* (e.g., Leber & Egeth, 2006). Assuming that selecting appropriate actions in pursuit of one’s goals usually requires selecting the “right” stimulus information, implementing an appropriate attentional set may be a crucial capability regarding successful goal achievement. It is also widely assumed that attentional sets can be adjusted, within certain boundaries, by intention (or “endogenously” or “top-down”),^1^ such as when the accomplishment of a novel goal requires (or would be facilitated by) the processing of hitherto irrelevant stimulus information for action control. Subjective impressions of switching one’s attention at will —such as becoming (more) aware of one of several speech messages at the expense of perceiving the others after deciding to engage in a conversation with a particular partner at a cocktail party— align with these conjectures. Successful task performance when participants are instructed to select their response based on a different stimulus feature than on a directly preceding trial in a laboratory experiment (for instance, when a visual search demands evaluating the presence of a target stimulus defined by different features on consecutive trials, e.g., Theeuwes & Van der Burg, 2008) is also consistent with them.

To achieve experimental evidence for intention-based changes of the attentional set, intention-unrelated factors that might influence the attentional set must be carefully controlled, however. In particular, changes of the attentional set might be brought about by bottom-up priming. For instance, facilitated responding to a stimulus presented at a particular location after perceiving a stimulus event taking place at that location (i.e., the standard finding in exogenous spatial cueing procedures, Posner & Cohen, 1984), may result from an attentional set featuring an enhanced representation of stimuli occurring at the location where the previous stimulus was perceived independently of a corresponding intention to do so. Various aspects of “stimulus saliency” have been discussed as factors that might bias stimulus selection (i.e., the attentional set) independently of current intentions (e.g., abrupt stimulus onset, Yantis & Jonides, 1984, or singular dissimilarity with other items in the display, Theeuwes, 2004).

To achieve reliable experimental evidence for intention-based changes of the attentional set, bottom-up priming must therefore be controlled. That is, trials for which advantageous responding is attributed to top-down attentional selection must not also be associated with perceptual conditions assumed to prime target processing in a bottom-up manner, such as high saliency of a stimulus event associated with the target or response (see Theeuwes, 2013, for a comprehensive overview and critique of experimental procedures). In this connection, it is also conceivable that having learned to increase the attentional weight of a particular stimulus feature on (a) previous trial(s)—for instance, because this feature used to be associated with target stimulus information in the past—may incur a persisting bias of processing stimulus information associated with this feature (i.e., a tendency of re-applying a corresponding attentional weight), independently of current intentions. Awh et al. (2012) refer to a general concept of *selection history* (“…past selection episodes are recapitulated in subsequent trials when the relevant context is encountered again”, p. 440) as a determinant of the attentional set. Contingencies of cues and target features likely play a role in this connection. For instance, a location may receive high activation after the presentation of a particular cue if it was previously experienced that following this cue, the target stimulus occurs with high probability in that location.

The latter type of contingency is usually implemented in studies that yield an “endogenous” spatial cueing effect (Posner, 1980). Although it seems likely that such contingencies are exploited intentionally (i.e., that activation of the location results from adjusting the attentional set according to the expected target location), it is also conceivable that merely experiencing contingencies leads to the formation of associations between cues and attentional responses previously made to the target, resulting in “automatic” adoption of a previously implemented attentional set when the cue is perceived. To illustrate, if an arrow cue pointing to the left is followed more frequently by a subsequent target on the left than on the right, whereas an arrow cue pointing to the right is frequently followed by a subsequent target to the right and vice versa, shifting the attentional set in response to the cue may be attributed to an acquired tendency of directing attention to a location that has been frequently experienced to involve relevant stimulus information in the past, even without an intention to do so. To achieve experimental evidence for intention-based changes in the attentional set, corresponding changes in the contingency between a cue and the perceptual feature entailing the target information should thus be avoided.

Shifts of the attentional set may play an important role in task switching. In such studies, participants are presented with two or more tasks, associated with at least partly divergent stimulus–response mappings, in varying sequence (for an overview, see Kiesel et al., 2010). Often, tasks differ regarding the perceptual target features, such as when participants are instructed to switch between identifying the color vs. the shape of colored shape stimuli. In these cases, successful task switching performance could theoretically be accomplished by a corresponding change of the attentional set (e.g., if one assumes constant stimulus–response links for both tasks and dominance of the currently relevant task in response selection to be ensured by applying larger attentional weight to the perceptual target features of this task than to the perceptual target features of the other task). Particularly convincing evidence for this conjecture comes from studies in which relevant stimulus information for the tasks was presented at different locations and gaze direction was used as a measure of attentional prioritizing. For example, Longman et al. (2013) recorded eye movements when participants switched between categorizing the photograph of a face or responded to a letter, superimposed on the face’s forehead. Although eye fixations on regions relevant for the currently irrelevant task occurred more frequently in task switch than in task repetition trials, this difference was reduced when the preparation interval was increased, suggesting preparatory adjustment of visual attention. Longman et al. (2014) even demonstrated adjustment of task-specific attentional sets during task preparation. Using three different tasks, comprising digit stimuli presented at task-specific locations, they observed orienting to the location relevant for the upcoming task (again slowed on task switch trials) prior to the presentation of the digits. Spatial orienting during the preparation interval was also observed when the location of the target digit varied among the three locations but the task was held constant.

In task switching situations, task cues usually do not bear any contingency relation with the occurrence of individual perceptual features as the same set of stimuli is used for both tasks and the same presentation likelihood applies to all stimuli regardless of the current task. By the same token, the effects of stimulus saliency or selection history could easily be dismissed as an alternative interpretation to as intention-based adjustment of the attentional set when switching between tasks.

Tasks differ concerning additional aspects, however, most notably regarding stimulus-specific response requirements. Indeed, we know of no task switching experiment in the extant literature in which not at least a subset of stimulus–response mappings differs between the tasks. That is, at least some of the stimuli are assigned a different motor response in one task than in the other—stimuli usually referred to as (response-) *incongruent* stimuli. Although task switching experiments frequently comprise a different subset of *congruent* stimuli (e.g., a red circle when one of the tasks requires pressing key A for red and key B for green, and the other task requires pressing key A for circles and key B for squares), confining the stimulus set to such congruent stimuli would seem to make it likely that participants would refrain from task switching and maintain only one of the tasks. Furthermore, lacking additional indicators for task-specific processing, deciding which task a participant executed on a given trial would be impossible. Therefore, changes in the attentional set that take place on task switch trials (if they do) are associated with foreseeable changes concerning stimulus–response mappings (at least in a subset of trials).

For purely intention-based adoption of an attentional set, we would assume that it could be realized in the absence of all additional changes in the task/stimulus environment (i.e., when task stimuli, responses, task-defined stimulus–response assignments, and stimulus-related response contingencies are kept constant). That is, one could, for instance, decide to “attend to” one of two simultaneously present messages, irrespective of the precise current response requirements (and even in the absence of any requirement of overt responding). If our analysis is correct, we currently have hardly any experimental evidence for this ability. In fact, the only experimental effect ascribed to a shift of the attentional set in the absence of *any change in stimulus-related contingencies or response requirements* we are aware of comes from a study of endogenous spatial cueing (i.e., shifting location-related attentional weights). Instructing participants to attend to location-unpredictive central cues (i.e., centrally presented arrows pointing randomly at one of eight possible target locations), Jonides (1981, Experiment 2) observed a cue validity effect of 61 ms (i.e., faster responding to a target presented at the cued than at an uncued location).^2^

Whereas it appears obvious that confounds of (bottom-up) priming by stimulus saliency or past selection history must be dismissed if one wants to infer intention-based shifts of the attentional set, the need for controlling stimulus-related response contingencies is less obvious. In principle, the fact that an attentional set is adjusted under conditions associated with a change in the instructed stimulus–response mapping clearly does not compromise the notion that the adjustment was brought about in an intention-based manner. Also, there seems to be no apparent reason to assume that intention-based adjustments made in such situations could not also be made in the absence of the mapping change. Nevertheless, experimental evidence might be valuable not only for the principled demand of supporting theoretical assumptions by “objective” facts. For one thing, putative evidence of particular instances of top-down adjustment of the attentional set has been doubted in various cases. For example, this skepticism has been raised in the domains of feature-based attention (Theeuwes, 2013) or conflict-related attentional focusing (Rünger et al., 2010; Wühr & Kunde, 2008). For another caveat, even if it is not seriously considered that additional changes in the task/stimulus environment might constitute a *necessary condition* for initiating shifts of the attentional set, ease of shifting may still be influenced by factors such as altered response requirements. That is, it might be easier to shift one’s attentional set if this is helpful to meet altered task demands or if it can be accomplished as part of more extensive task-set reconfiguration. The availability of experimental protocols allowing the assessment of changes in the attentional set associated with variations of additional shifting requirements, such as stimulus–response assignments of different compatibility or familiarity, would help achieve a comprehensive picture concerning conditions favoring shifts of the attentional set. Finally, assuming attentional sets of different efficiency for a given task would allow investigation of determinants of set selection other than ensuring optimal performance.

In this article, we present a procedure designed to investigate instructed shifts of the attentional set in the absence of additional changes concerning target and distractor stimuli, stimulus-related contingencies, or response demands. The procedure can be described as a task switching protocol involving only congruent stimuli. Although such a procedure does not allow determining whether a task switch was made (because each correct or incorrect response in the context of one of the tasks is equivalently correct or incorrect in the context of the other task), it appears possible to assess shifts of the attentional set by intermixing trials of another (probe) task (e.g., Kähler et al., 2020, 2024; LaBerge, 1983; Wendt et al., 2012, 2014, 2017). Our point of departure pertains to evidence obtained with this probe task methodology for shifts of the attentional set as a function of cueing tasks that feature different demands of stimulus selection (as well as different stimulus–response mappings). More precisely, Wendt et al. (2017) and Kähler et al. (2020) presented participants with two tasks in which a string of three letters had to be categorized either according to the identity of the central letter (Eriksen task, assumed to evoke narrowing the focus of attention to a small central region) or according to a “global” property of the string (homogeneity/heterogeneity, Wendt et al., 2017, or symmetry, Kähler et al., 2020, assumed to be associated with widening the attentional focus). The two tasks were presented in random sequences and indicated by a task cue preceding the letter string to allow task preparation and instruct the actual upcoming task. Occasionally, however, a visual search task^3^ was presented, in which a to-be-identified target stimulus could occur at any of the three letter locations. Search task results displayed a more pronounced center-to-periphery gradient of search times, indicating stronger attentional focusing on the central position after a cue signaling the Eriksen task than after a cue signaling the global judgment task.

In another series of experiments, Wendt et al. (2014) combined the visual search task with a modified Eriksen flanker task in which, on each trial, a centrally presented target letter was flanked by two copies of the same letter on either the left or right side and by two copies of another letter, assigned a different response, on the opposite side (e.g., *HHHSS* or *HHSSS*, target *H* and *S* requiring a left-sided and a right-sided key press, respectively). With this asymmetric flanking procedure, the letter presented in the center always occurred three times in a given display. This procedure allowed the authors to instruct two different groups of participants to respond to the “central letter” vs. to the “letter presented three times”, respectively, keeping all other task parameters identical (i.e., stimuli, responses, stimulus–response mappings, and presentation procedures). Intermixed trials of a visual search task yielded a more pronounced center-to-periphery search time gradient in the group of participants instructed to respond to the central letter. This pattern was considered evidence for stronger focusing of attention at the central letter position in the central instruction group than in the group instructed to respond to the letter presented three times.

In the current study, we combined the two approaches of instructing switches between two “tasks” associated with different demands of stimulus selection (and intermixing trials of a visual search task to assess the attentional set as a function of the instruction) and keeping the tasks constant with respect to all stimulus/response features by using asymmetrical three-letter strings in which the central letter was always presented more frequently (i.e., two times) than a distractor letter (i.e., one time). We reasoned that if instructing participants to respond to the “central letter” vs. the “letter presented twice” (henceforth “center instruction” and “double instruction”, respectively) results in corresponding shifts of the attentional set, we would observe a more pronounced center-to-periphery search time gradient in the former than in the latter case. In the between-groups manipulation applied by Wendt et al. (2014), it might not have been obvious to participants of a given group that the instructed task could be completed equivalently by the other strategy (because the other strategy was never mentioned to any participant). Conversely, in the current study, it was obvious to all participants that the instructed switch in attentional strategy was unnecessary to perform the task because both strategies would result in the same (correct) motor response. Not only were both strategies introduced during the practice trials, but each participant was explicitly told beforehand that both strategies would lead to identical responses (except for Experiment 1, in which only one instructional switch was implemented halfway through the experimental session; thus, the second strategy was unknown in the first block).

In the flanker task of our experiments, the letters A and B were mapped to one response key and the letters C and D were mapped to another response key. With this arrangement the single letter of a given stimulus string that served as a distractor could either be associated with the same response as the target (e.g., AAB, CDD; henceforth referred to as *congruent*) or with a different response (e.g., AAC, DBB; henceforth referred to as *incongruent*). Assessing the congruency effect (i.e., the performance difference in congruent and incongruent trials) provided us with information concerning the amount of distractor-based interference. This allowed us to examine whether distractors exert lower impact on performance when participants are instructed to respond to the central letter, which would be consistent with the assumption of adopting a narrower focus of visual attention.

## Experiment 1

In our first experiment, participants were instructed to switch their attentional strategy only once. More precisely, one group of participants started with the center instruction whereas a different group started with the double instruction. After completing half of the trials of the experimental session, they were informed about the other strategy and instructed to use it until the end of the experimental session.

If participants adhered to instructions, we expected them to implement a narrower focus of visual attention under the center instruction than under the double instruction. Although instruction-based adjustment of the attentional set might evidence itself in flanker task performance (i.e., smaller congruency effect with center instruction), our primary measure for this assessment was the search time gradient in the probe task. Precisely, we expected to observe a more pronounced center-to-periphery gradient of search times under the center instruction than under the double instruction (resembling the findings obtained for groups of participants receiving different instructions in Wendt et al., 2014, Experiment 1).

### Method

The methods employed in the four experiments presented were similar and are described in detail in this section. Any deviations from this description are noted in the respective sections of each individual experiment.

#### Participants

Fifteen male and five female participants (aged 21 to 28, *mean* = 23.80, *SD* = 1.85) of the Helmut Schmidt University/University of the Federal Armed Forces Hamburg either to fulfill course requirements or without reward. All participants in the four studies gave informed consent prior the experiments.

#### Apparatus and stimuli

All four experiments were very similar, using the same hardware, stimuli, responses, etc., so a general description is given here. The main differences were in the instructions, task cues, number of experimental blocks, and questions about the participants’ strategy; the differences between experiments follow this general description.

The experiments were run in a room with six identically constructed cabins (internal dimensions: width = 120 cm, depth = 180 cm, height = 205 cm), ventilated, lighted and noise-dampened. The window in the box was not covered. The experiments were run under MS-DOS 6.11 and programmed with Borland’s Turbo C + + 3.0. They were displayed on an AOC 919 19-inch LCD Screen (refresh rate 60 Hz, latency 2 ms), and responses were made with a gaming keyboard (“Storm Quickfire Pro”, Cooler Master, latency 1 ms), with the response keys placed approximately 35 cm centrally in front of the screen. The participants sat approximately 60 cm in front of the screen and executed the task by pressing “v” (“left response key”) with their left hand or “n” (“right response key”) with their right hand, both with either the index- or middle finger.

Two tasks were performed, the flanker task (letters) and the probe task (digits). Flanker and probe trials were presented with different probabilities depending on the type of preceding trial. After a flanker trial (or the start of a new block of trials), another flanker trial was presented with a probability of 8/11, and a probe trial was presented with a probability of 3/11. After each probe trial, a flanker trial always followed. These probabilities produced frequencies of 78.51% flanker trials and 21.49% probe trials. The frequency for the flanker task was higher than 8/11 (72.27%), %), because consecutive probe trials were excluded. Apart from these constraints, all stimuli were chosen randomly.

The stimulus material was presented on a medium gray background within a white frame (78 × 78 mm). For both tasks (flanker and probe), the stimulus contains three vertically arranged elements, each element extended between 5 and 10 mm horizontally and 12 mm vertically. The whole string extended approximately 0.7° horizontally and 3.8° vertically. In the flanker task, a central letter (*A*, *B*, *C,* or *D*) was surrounded asymmetrically: on one side the same letter, and on the other, a different letter. The letters were mapped to responses in a 4:2 manner (*A*, *B* left key; *C*, *D* right key). Participants were instructed to either respond to the central letter (center instruction), or the letter presented twice (double instruction), although both instructions lead to the same response since the central letter was always the same as the letter presented twice. The protocol of switching instructions is described in the procedures for each experiment.

The probe task digit string, with the same three positions for the stimulus elements as in the flanker task, was made of three digits. The target was either a “3” (left response) or a “7” (right response), chosen at random and presented at random at the top, center, or bottom of the string. The remaining two positions were filled with two random digits from 1 to 9, with the constrains that no digit could appear twice in the same trial and only one target was present.

#### Procedure

**Fig. 1:**
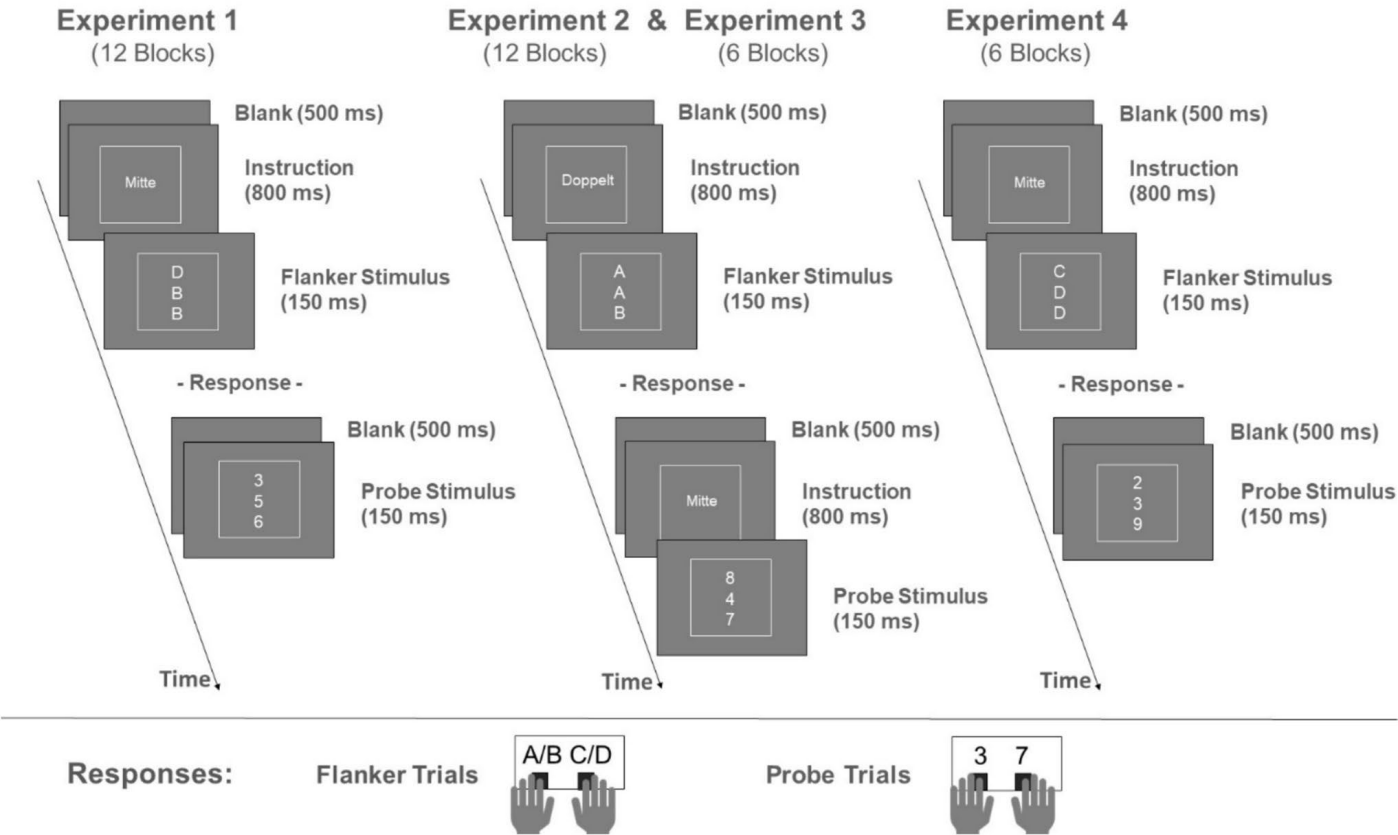
Schematic representation of the sequence for flanker and probe trials in Experiments 1 to 4, incorporating either a “Center” (*Mitte*) instruction or a “Double” (*Doppelt*) instruction. In Experiment 1, the instructions were switched once after half of the blocks, whereas in Experiments 2, 3, and 4, the instructions were switched on a trial-to-trial basis

Each trial started immediately after the previous response with a period of 500 ms showing a blank inside the frame. Immediately after, an 800 ms period followed, during which either a task cue or a blank was shown (except in Experiment 1, see below). Finally, the imperative stimulus was presented for 150 ms only, and the program waited until a response was given (see Fig. 1). The two cues used were the words “Mitte” (German for “center”, approx. 1.9° wide) and “Doppelt” (German for “double”, approx. 2.9° wide). If the response was incorrect, the German word for “false” (“falsch”) was presented for 800 ms below the stimulus on the bottom of the frame, and the trial was repeated but not stored. Any response other than “v” or “n” produced the message “unrecognized key”.

The general procedure started with instructions for the probe task, followed by a 20-trial practice block. Then, instructions for the flanker task were given, followed by a 40-trial practice block consisting of flanker and probe trials in the same proportion as in the experimental blocks. Each block consisted of 99 trials, discounting repetitions after errors. After each experimental block, feedback was given, including the average reaction time and percentage of errors, and participants could make a pause. The number of experimental blocks varied depending on the experiment.

In Experiment 1, each flanker trial featured a cue, as described above. However, probe trials were presented 500 ms after the last response without a cue. Twelve experimental blocks were performed; in the first six blocks, only one of the cues was used (also in the mixed-task practice block). The new cue and instructions on how to perform the task from now on were introduced after the sixth block, and the other cue was used in the final six blocks. The order of cues was balanced across participants. Afterward, participants were asked if they could follow the second instructions. An overall experimental session took about 45 min.

### Results

In all analyses detailed in this manuscript, we employed the following standards. The initial practice blocks and the opening three trials of each experimental block were classified as warm-up trials. These, thus, were omitted from the analysis (for RT and error rates). Regarding RTs, trials featuring the correct response falling within a range of 2.5 standard deviations around the mean of each participant's responses to either the probe or flanker task, with an added threshold set at a minimum of 200 ms, were scrutinized. Erroneous responses and the subsequent trial were disregarded in the RT data analysis. For the analysis of probe trials, this resulted in a data loss of 18.59% for RTs and 5.55% for error rates. For the analysis of flanker trials, this resulted in a data loss of 18.71% for RTs and 6.24% for error rates. The results presented are corrected using the Greenhouse–Geisser method and were computed with R (R Studio Team, 2022.07.02), which was also used to prepare of the result figures.

To determine the minimum detectable effect size for the within effects, a sensitivity analysis was conducted using G*Power Version 3.1.9.4 (Faul et al., 2007). For probe tasks of the experiments, the sensitivity analysis (*N* = 20, *p* = 0.05, β = 0.8, repeated measures = 3) showed large-sized effects to be detectable, based on the given sample size (F(2, 38) = 3.24, ηp^2^ = 0.15). For the flanker tasks of the experiments, the sensitivity analysis (*N* = 20, *p* = 0.05, β = 0.8, repeated measures = 2) showed large-sized effects to be detectable, based on the given sample size (F(1,19) = 4.38, ηp^2^ = 0.19).

To corroborate all interaction effects of theoretical relevance, we computed inclusion or exclusion Bayes factors (depending on the direction of empirical effect). Van den Bergh et al. (2020) derived these Bayes factors from comparisons between matched models, meaning that every model incorporating the effect of interest is tested against all models that exclude it. An inclusion factor (BF_incl_) of X suggests that the data are X times more probable under models that contain the effect, whereas its reciprocal (BF_excl_ = Y) indicates that the data are Y times more probable under models omitting the effect. Bayesian analyses were conducted using JASP (Version 0.17.3.0), with default settings of r(fixed effects) = 0.05, r(random effects) = 1.0, r(covariates) = 0.354, and 10,000 iterations. Bayes Factors for the planned comparison of quadratic trends were calculated from the weighted Data of target positions (1/2 for the top and bottom position, each).

#### Probe task

**Fig. 2:**
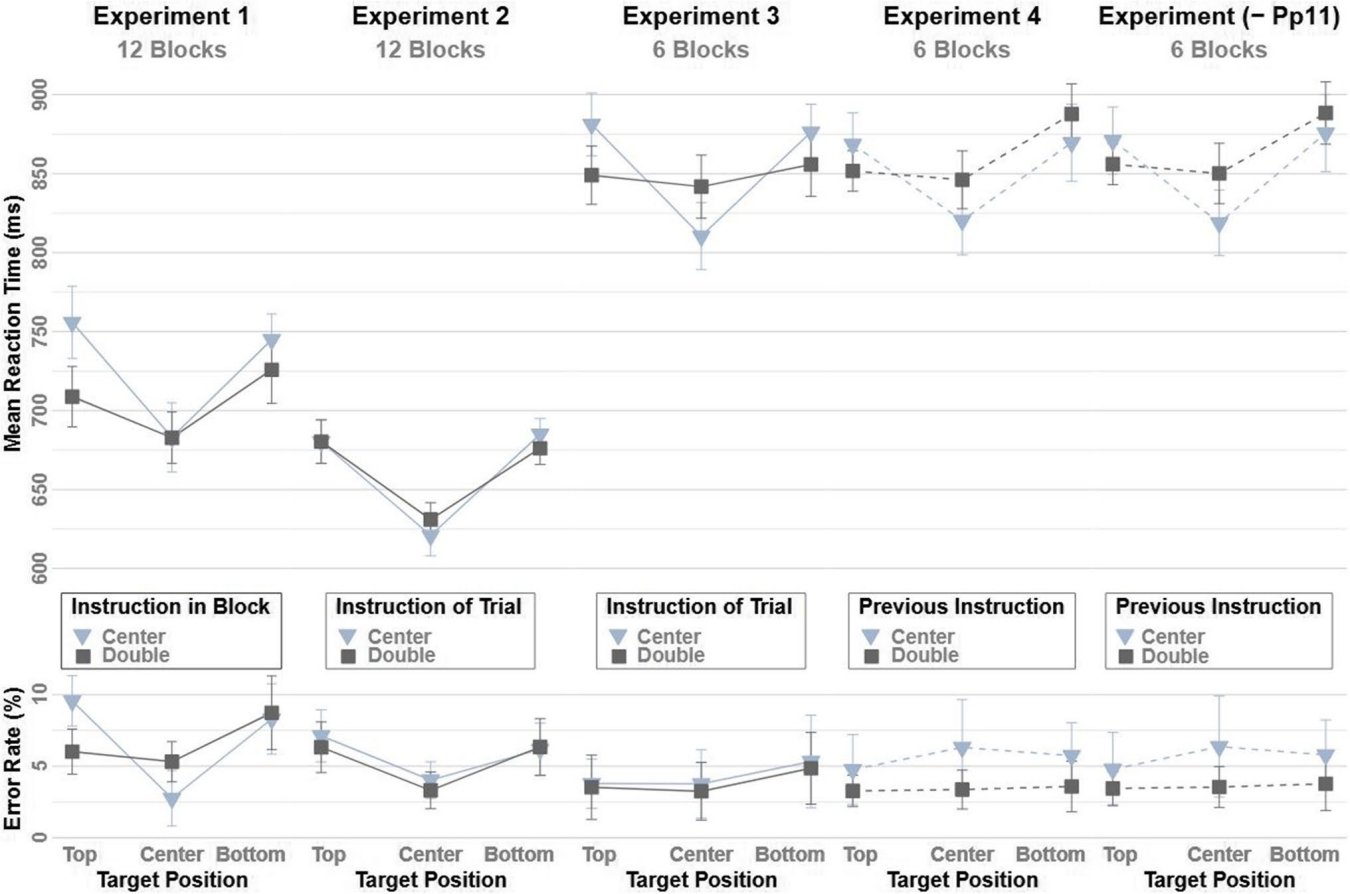
Mean reaction times and error rates in the probe-task of Experiment 1–4 as a function of Instruction in Block/Instruction/Previous Instruction (Center, Double) and Target Position (Top, Center, Bottom). Error bars reflect the 95% within-confidence Intervals. -Pp11 = Participant 11 excluded

A 2 × 2 × 3 mixed ANOVA with repeated measures on the within-subjects factors Instruction (Center, Double), Target Position (Top, Center, Bottom), and the between-subjects factor Start-Condition (Center, Double) was run on the mean RTs. The main effect of Target Position F(1.97, 35.458) = 28.611, *p* < 0.001, *ηp*^*2*^ = 0.614 reached significance due to facilitated responses for probe targets presented at the center (683 ms) compared to the top (732 ms) and bottom (735 ms) position. The two-way interaction of Start Condition × Instruction *F*(1, 18) = 6.246, *p* = 0.022, *ηp*^*2*^ = 0.258 reached significance, as well as the two-way interaction of Instruction × Target Position *F*(1.965, 35.373) = 7.677, *p* = 0.002, *ηp*^*2*^ = 0.299. The former was due to slower responses for the two group’s first block (*StartCenter*_*BlockCenter*_ = 732 ms, *StartDouble*_*BlockDouble*_ = 731 ms) compared to the group’s second block (*StartCenter*_*BlockDouble*_ = 681 ms, *StartDouble*_*BlockCenter*_ = 724 ms). The latter displayed a steeper gradient of search times for blocks with the center instruction (*Top* = 756 ms, *Center* = 683 ms, *Bottom* = 745 ms) compared to the block with the double instruction (*Top* = 709 ms, *Center* = 683 ms, *Bottom* = 726 ms). Planned comparison of the quadratic trends over the three target positions confirmed this modulation of the center-to-periphery gradients, more precisely, the interaction of Instruction × Target Position *F*(1, 18) = 9.432, *p* = 0.007, *ηp*^*2*^ = 0.344 (see Fig. 2). The Bayesian analysis for the interaction of Instruction × Target Position and for corresponding quadratic trends produced very strong (BF_incl_ = 38.197 and strong (BF_incl_ = 11.929) evidence, respectively, in favor of the effect.

An equivalent ANOVA for the mean error rates displayed a significant main effect of Target Position *F*(1.744, 31.392) = 11.6, *p* < 0.001, *ηp*^*2*^ = 0.392, with less error-responses to the central- compared to the peripheral positions (*Top* = 7.78%, *Center* = 4.03%, *Bottom* = 8.50%) and a significant two-way interaction effect of Instruction × Target Position *F*(1.988, 35.783) = 6.22, *p* = 0.005, *ηp*^*2*^ = 0.25. A steeper gradient of error rate for blocks with the center instruction (*Top* = 9.55%, *Center* = 2.75%, *Bottom* = 8.29%) compared to the block with the double instruction (*Top* = 6.01%, *Center* = 5.31%, *Bottom* = 8.72%). Planned comparison of the quadratic trends over the three target positions confirmed this modulation of the center-to-periphery gradients, more precisely, the interaction of Instruction × Target Position *F*(1, 18) = 7.379, *p* = 0.014, *ηp*^*2*^ = 0.291. The Bayesian analysis for the interaction of Instruction × Target Position and for corresponding quadratic trends produced very strong (BF_incl_ = 30.379 and strong (BF_incl_ = 12.653) evidence, respectively, in favor of the effect.

No other effect reached significance; for full results see Appendix 2.

#### Flanker task

A 2 × 2 × 2 ANOVA with repeated measures on the factors Congruency (Congruent, Incongruent), Previous Congruency (Congruent, Incongruent), and Instruction (Center, Double) was run on the mean RTs and error rates of the flanker task. The main effect of Congruency reached the level of significance *F*(1, 19) = 13.006, *p* = 0.002, *ηp*^*2*^ = 0.406 as congruent trials were answered faster than incongruent trials (*Congruent* = 489 ms, *Incongruent* = 495 ms). Likewise, the main effect of Instruction *F*(1, 19) = 12.844, *p* = 0.002, *ηp*^*2*^ = 0.403, due to, on average, facilitated responses following the center instruction (479 ms) compared to the block with the double instruction (505 ms). The Bayesian analysis for the null effect of Instruction × Congruency yielded moderate evidence against the interaction effect (BF_excl_ = 3.504).

In the analogous ANOVA for the error rates, no effects reached the level of significance, even though flanker interference tended to be lower in the block with the center instruction (*Congruent* = 5.40%, *Incongruent* = 5.39%) compared with double instruction blocks (*Congruent* = 5.26%, *Incongruent* = 7.05%) *F*(1, 19) = 3.178, *p* = 0.091, *ηp*^*2*^ = 0.143. However, the Bayesian analysis for the effect of Congruency × Previous Congruency yielded moderate evidence against the interaction effect (BF_excl_ = 3.746). The Bayesian analysis for the null effect of Instruction × Congruency interaction yielded only anecdotal evidence against the interaction effect (BF_excl_ = 1.041).

**Fig. 3:**
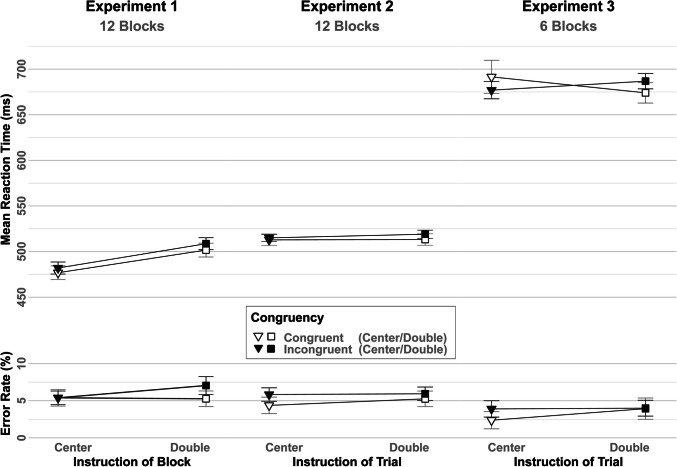
Mean reaction times and error rates in the flanker-task of Experiment 1–3 as a function of Instruction (Center, Double) and Congruency (Congruent, Incongruent). Error bars reflect the 95% within-confidence Intervals

Full results are presented in Fig. 3 and Appendix 2.

### Discussion Experiment 1

Experiment 1 yielded a more pronounced center-to-periphery search time gradient of probe trials in blocks of flanker trials in which participants were presented the center instruction than in blocks of trials in which participants were presented the double instruction. This result is consistent with the assumption that participants shifted their attentional set according to instruction from a stronger to a lesser degree of focusing visual attention or vice versa, depending on their starting condition. This shift of the attentional set occurred despite the fact that—with the exception of the cue that reminded the participants of the to-be-applied attentional strategy—all stimuli, responses, stimulus–response mappings, and stimulus-related contingencies were identical for the two instruction conditions. The results of Experiment 1 thus extend the findings of Wendt et al., (2014, Experiment 1) in which adherence to the instruction to respond to the “central letter” vs. to the “letter that occurred three times” in the display was found for two different groups of participants. In that situation, adherence to instruction is not surprising because participants might not have been aware of the other possible strategy.

Interestingly, flanker task performance was overall better (i.e., responses were faster) under the center instruction than under the double instruction, suggesting that focusing attention on the central letter constitutes an overall superior strategy. If this is the case, participants of the group that started with the center instruction shifted their attentional set as instructed although this resulted in disadvantageous processing.

Trials featuring an incongruent flanker yielded longer RTs than trials featuring a congruent flanker. Despite faster responding under the center instruction overall, flanker interference was not significantly lower than under the double instruction (except for a non-significant trend in error rates). This suggests that the search time gradient of our probe task was more sensitive for the degree of focusing of visual attention than the flanker congruency effect. Although the overall magnitude of the flanker congruency effect was small (i.e., 6 ms, however, supported a large effect size and strong evidence of the Bayesian Analysis), this is not surprising because only one of the two flankers differed from the central letter. Thus, processing impairment in incongruent trials was based exclusively on response conflict unconfounded with “stimulus conflict” (i.e., the deviant flanker was associated with a different vs. with the same response as the target in incongruent and congruent trials, respectively, whereas congruent trials were not characterized by perceptual identity of all constituent elements of the letter string as is frequently the case in flanker task studies).

## Experiment 2

Having established the general feasibility of the probe task method to detect differences in the degree of attentional focusing applied to asymmetrically flanked letter stimuli under different instructions (i.e., “respond to the central letter” vs. “respond to the letter presented twice”) we set out to investigate the degree of flexibility with which participants can (and are willing to) shift their attentional set despite being aware of the equivalence of the two strategies with regard to selecting the required response. More specifically, in Experiment 2 the instructional cue varied randomly from trial to trial. Participants were thus asked to respond to the centrally presented letter or to the letter presented twice in randomly chosen 50% of the trials each. Assuming that adhering to instructions results in adopting different attentional sets (i.e., narrower or broader focus of visual attention) implies that the attentional set would be frequently shifted during the course of the experimental session. Obtaining evidence for this assumption in the form of a steeper search time gradient in probe task trials featuring the center instruction than in probe task trials featuring the double instruction would extend the findings of Experiment 1 which testify to a single shift of the attentional set, occurring at some point during the second phase of the experimental session, possibly after multiple reminders to do so by the altered cue. Random choice of the cue in each trial provides us with the possibility of examining the role of “repeated reminders” by comparing the search time gradient in trials in which the instructional cue switched from the preceding trial and in trials in which the cue repeated.

Comparing the search task gradient for trials with repetition and switch of the instructional cue is of interest from another point of view. Precisely, if the attentional set is adapted on the basis of a trial’s cue, then analyzing the search time gradient as a function of both the cue of the current trial and the cue of the preceding trial would allow us to assess the interplay of cue-based alteration of the attentional set and attentional carryover from the preceding trial. Although attentional carryover has been reported in various tasks (e.g., Belopolsky et al., 2010; Müller et al., 1995; Longman et al., 2013; Longman et al., 2014; Theeuwes & Van der Burg, 2011), previous applications of our probe task method, requiring frequent switches between tasks associated with strong vs. weak focusing of attention (i.e., Kähler et al., 2020; Wendt et al., 2017) yielded no evidence for carry over effects of the attentional set adopted on the preceding trial. As mentioned in the Introduction, these studies demonstrated a steeper search time gradient in trials in which the cue indicated a task requiring attentional focusing on a central stimulus location than in trials in which the cue indicated a task requiring a judgment concerning the complete stimulus configuration. By contrast, attentional carryover (i.e., steeper search time gradients in trials following the “focusing task” than following the “global judgment task”) was only observed when cue-based preparation in probe task trials was prevented (by presenting the imperative stimulus at the time the cue was expected to occur; Kähler et al., 2024, Experiments 2 A and 2B). These findings agree with the notion of complete replacement of the previous attentional set during task preparation. Assuming that a similar replacement of the attentional set would occur in Experiment 2, we expected that the center-to-periphery gradient of search times in the probe task would be more pronounced in trials featuring the center instruction than in trials featuring the double instruction. By contrast, it should not be affected by the instruction provided on the preceding (flanker task) trial.

### Method

For the general apparatus, stimuli, and procedure see Sect."Method". All changes are outlined below.

#### Participants

Twenty students, with a mean age of 23.55 (*SD* = 2.44; *Range* = 19–27; 10 males, 10 females), and the same background and reward as in Experiment 1, participated in Experiment 2.

#### Apparatus & stimuli

Apparatus and Stimuli were the same as described in 2.1.2.

#### Procedure

In this experiment, the cue (i.e., “center” or “double”) was chosen randomly on each trial, including the probe trials. It should be noted, that after a “center” cue, a probe target positioned on the top or bottom of the string can lead to a degraded performance. In other words, the cue can be misleading in probe trials.

After completing the experiment, participants were interviewed regarding whether they could adjust their attention according to the cues. An overall session took between 45 and 60 min.

### Results

The data exclusion and calculation of the experimental data were conducted as described in Experiment 1. For the analysis of probe trials, this resulted in a data loss of 18.33% for RTs and 6.19% for error rates. For the analysis of flanker trials, this resulted in a data loss of 17.82% for RTs and 5.86% for error rates.

#### Probe task

A 2 × 2 × 3 ANOVA with repeated measures on the factors Instruction (Center, Double), Previous Instruction (Center, Double), and Target Position (Top, Center, Bottom) was run on the mean RTs and error rates. Only the main effect of Target Position reached significance *F*(1.915, 36.387) = 44.503, *p* < 0.001, *ηp*^*2*^ = 0.701, due to facilitated responses for probe-targets presented at the center (626 ms) compared to the top (680 ms) and bottom (681 ms) positions. The Bayesian analysis for the null effect of the interaction of Instruction × Target Position and for corresponding quadratic trends produced moderate (BF_excl_ = 3.266) and anecdotal (BF_excl_ = 1.295) evidence, respectively, against the effect. An equivalent ANOVA for the mean error rates displayed a significant main effect of Target Position *F*(1.781, 33.832) = 8.493, *p* = 0.001, *ηp*^*2*^ = 0.309, with fewer errors for targets presented at the center (3.67%) compared to the top (6.72%) and bottom (6.25%) positions. The Bayesian analysis for the null effect of the interaction of Instruction × Target Position and for corresponding quadratic trends produced moderate evidence (BF_excl_ = 7.905 and BF_excl_ = 3.447, respectively) against the effect. No other effect reached the level of significance.

For results, see Fig. 2 and full results are displayed in Appendix 2.

#### Flanker task

A corresponding 2 × 2 × 2 ANOVA with repeated measures on the factors Congruency (Congruent, Incongruent), Previous Congruency (Congruent, Incongruent), and Instruction (Center, Double) was run on the mean RTs and the error rates. The RTs yielded no significant effects. The Bayesian analysis for the null effect of Instruction × Congruency yielded only anecdotal evidence against the interaction effect (BF_excl_ = 2.646). Only in the error rates, the main effect of Congruency reached significance *F*(1, 19) = 4.528, *p* = 0.047, *ηp*^*2*^ = 0.192, with reduced error rates in congruent trials (4.81%) compared to incongruent trials (5.89%). The Bayesian analysis for the null effect of Instruction × Congruency yielded only anecdotal evidence against the interaction effect (BF_excl_ = 2.385).

For results, see Fig. 3, and full results are displayed in Appendix 2.

#### Adherence to instructions

In the verbal interview following the experiment, eight participants indicated they followed the instructions, whereas ten reported, they stopped the adherence to instructions throughout the experiment. Two described the switch as “automatic”.

### Discussion Experiment 2

Unlike Experiment 1 (i.e., block-wise adherence to instruction), search times were unaffected by the trial’s instruction, failing to provide evidence for instruction-based adjustment of the attentional set on a trial-by-trial basis. Consistent with this latter finding, we did not observe any carryover effects from the preceding trial, and at least 50% of participants reported eventually stopping the switch between instructions. Concerning the flanker task, we observed a congruency effect in the error rate, which was not modulated, however, by the current or the preceding instruction.

The reasons for non-adherence to the instructions among the participants, or at least 50% of them, remain unclear. As an alternative to the radical view that the participants were simply not capable of adopting the instructed (but unnecessary) attentional adjustment from trial-to-trial, decreasing cognitive control (or increasing cognitive fatigue) or motivational factors (see Botvinick & Braver, 2015) could have impaired the ability and/or willingness to switch between the instructed attentional sets. Research on strategies to target cognitive fatigue has used methods such as breaks, shorter experiments/blocks, incentives, manipulation checks as an assessment of cognitive fatigue (or motivation) itself and as a basis for excluding data from trials associated with high fatigue (for discussions, see Botvinick & Braver, 2015; Behrens et al., 2023; Gilsoul et al., 2022; Gawron, 2014; Mackworth, 1948; Sarter et al., 2006; Teichner, 1974; Warm et al., 2008). As discussed above, because both instructions led to the same responses, failure to adhere to the instruction did not result in overtly erroneous responding (i.e., no measurable performance parameters). The approaches described for mitigating fatigue or motivational difficulties might enable the instructed adaptation of attentional sets through modifications in the experimental design.

## Experiment 3

In Experiment 3, the discussed methods for improving cognitive control and/or motivational factors suspected to be the cause of the null finding of Experiment 2 (i.e., search time gradients in the probe task that did not differ significantly as a function of the cue), were examined. We included two questions following each experimental block to assess participant’ subjective ratings of their degree of adherence to instructions. First, participants indicated their adherence to the instructions in the preceding block on a 4-point Likert-scale. This was followed by a question asking which instruction they followed more closely in the preceding block (“center”, “double” or “both to the same amount”). This process was not only intended as a manipulation check but also to allow participants to reflect on their adherence to instruction and potentially reinforcing its importance. In addition, we reduced the number of experimental blocks from 12 to six, which left around 40 trials of each probe-position-instruction combination from the diagnostic probe-task. In this way, we aimed to decrease the loss of cognitive control during the course of the session and collect data about self-assessed adherence to instruction as a criterion for selecting subsets of the data for post hoc analyses. A distinct criterion was not defined beforehand. One possible application of the additional data could be to exclude individual blocks with a specifically low (self-assessed) adherence to instruction from the data of a participant. However, this approach is problematic, as it would leave even fewer probe trials for the analysis. If only a small proportion of participants reported low adherence to instructions, another option would be to exclude their data from the analysis to explore its impact on the results.^4^

### Method

For the general apparatus, stimuli, and procedure, see Sect."Method". All changes are outlined below.

#### Participants

In Experiment 3 participated seven female and 13 male students with the same background as in Experiment 1. The mean age was 21.7 (*SD* = 2.2; *Range* = 19–25).

#### Apparatus & stimuli

Apparatus and Stimuli were identical, as described in 2.1.2.

#### Procedure

This experiment was identical to Experiment 2, with two exceptions. Only six experimental blocks were administered. After each block, participants were asked on-screen to report how frequently they actually followed the instructions (response options: 0–25%, 26–50%, 51–75%, 76–100%), followed by a question about which instruction they followed more frequently (response options: “center”, “double” or “both to the same amount”).

### Results

The data exclusion and calculation of the experimental data were conducted as described in Experiment 1. For the analysis of probe trials, this resulted in a data loss of 18.40% for RTs and 8.97% for error rates. For the analysis of flanker trials, this resulted in a data loss of 22.06% for RTs and 13.41% for error rates.

#### Adherence to instructions

As adherence to instructions in the six blocks was assessed in steps of 25% (see Sect."Procedure"), the values 1–4 were assigned to the categories to calculate the mean self-assessed adherence to instructions (see Table 1). Participant 17 did not produce valid data; however, the follow-up interview indicated adherence to instructions (see Sect."Follow-up interview of adherence to instructions"). The overall mean adherence to instructions was 3.44 with a SD of 0.74. None of the participants’ adherence to instructions fell outside the range of 2.5 SDs (as applied for trimming RTs) around the mean adherence to instructions (*range* = 1.59—5.29). The sample’s median self-assessed adherence to instructions was “75–100%” (*N* = 75), followed by 22 blocks with “51–75%”, and only nine and eight blocks of “26–50%” and “0–25%”, respectively.

**Table 1:** Self-assessed adherence to instructions by participants (PPs) in Experiment 3, following each experimental block

	PP	1	2	3	4	5	6	7	8	9	10	11	12	13	14	15	16	17	18	19	20
Block																					
1		3	4	4	4	4	4	4	4	1	4	4	4	4	4	4	4	NA	4	3	4
2		3	4	4	4	4	1	4	3	3	4	4	3	4	4	3	4	NA	4	4	3
3		2	4	4	4	4	1	4	3	2	3	4	3	4	4	4	3	NA	4	4	4
4		2	4	4	4	4	2	4	3	2	4	4	1	3	4	4	4	NA	4	1	4
5		1	4	4	4	4	2	4	3	3	3	4	1	4	4	3	4	NA	4	4	4
6		1	4	4	4	4	2	4	3	2	4	4	2	3	3	3	4	NA	4	4	4
mean		2	4	4	4	4	2	4	3.17	2.17	3.67	4	2.33	3.67	3.83	3.50	3.83	NA	4	3.33	3.83

Values were assigned to indicate perceived adherence to instructions as follows: 0–25% (1), 26–50% (2), 51–75% (3) and 76–100% (4) of trials in the block

The results for the strategies used following each experiment demonstrated that most participants primarily applied “both strategies about the same amount” (55.83%). This option aligns with the actual frequencies in the blocks and supports high compliance. The next most frequently applied strategy was “double” (24.17%), followed by “center” (14.17%). Missing data accounted for 5.83% (see Table 2). Overall, this pattern matches the high levels of compliance, as a nearly equal application of both strategies would indicate strong adherence. However, we did not further inquire about the exact frequency.

**Table 2:** Self-assessed strategies applied by participants (PPs) in Experiment 3, following each experimental block

Block	1	2	3	4	5	6	7	8	9	10		11	12	13	14	15	16	17	18	19	20
1	3	3	3	2	3	3	3	3	2	3		2	2	3	2	3	3	NA	3	2	3
2	3	3	3	1	2	3	3	2	1	3		1	2	3	3	3	2	NA	1	3	2
3	2	3	3	1	1	3	3	2	2	2		3	3	2	3	3	1	NA	3	3	3
4	1	3	3	1	1	3	2	2	2	3		3	2	3	1	3	3	NA	1	NA	3
5	2	3	3	1	2	3	3	2	2	2		3	NA	1	3	3	3	NA	1	3	3
6	2	3	3	1	3	3	3	2	2	3		3	2	1	3	3	3	3	3	3	3
N of blocks applied both strategies	2	6	6	0	2	6	5	1	0		4	4	1	3	4	6	4	1	3	4	5

Values were assigned to the predominantly applied strategy in the block (1 = “center”, 2 = “double” or 3 = “both to the same amount”)

The lowest compliance was observed among Participants 1, 9, and 12. They had low mean compliance scores (2.00, 2.17, and 2.33, respectively) and a low number of blocks in which they applied both strategies to about the same amount (two, zero, and one, respectively). Participants 6 and 8 followed, with Participant 6 having a low mean compliance of 2; however, in all blocks, both strategies were applied about the same amount (indicating adherence to instructions). Participant 8 had a higher mean compliance score (3.17); however, only one block with both strategies applied about the same amount. Finally, Participants 5 and 4 had mean compliance scores of 4 (corresponding to adherence in 75–100% of trials); however, both had relatively few blocks in which both strategies were applied about the same amount (two and one, respectively). Participant 17 did not produce valid data, except for the last block, where both strategies were applied about the same amount. In line with these data, the verbal interview following the experiment indicated high adherence to instructions.

#### Probe task

A 2 × 2 × 3 ANOVA with repeated measures on the factors Instruction (Center, Double), Previous Instruction (Center, Double) and Target Position (Top, Center, Bottom) was run on the mean RTs and error rates. The main effect of Target Position reached significance *F*(1.68, 31.922) = 8.835, *p* = 0.002, *ηp*^*2*^ = 0.317, indicating facilitated responses for probe-targets presented at the center (826 ms) compared to the top (865 ms) and bottom position (866 ms). The interaction of Instruction and Target Position reached the level of significance *F*(1.901, 36.126) = 6.463, *p* = 0.005, *ηp*^*2*^ = 0.254. This was confirmed by the quadratic trends, demonstrating a more pronounced center-to-periphery gradient *F*(1, 19) = 14.355, *p* = 0.001, *ηp*^*2*^ = 0.43 after the center instruction (*Top* = 881 ms, *Center* = 810 ms, *Bottom* = 876 ms) compared with that of the double instruction (*Top* = 849 ms, *Center* = 842 ms, *Bottom* = 856 ms). The Bayesian analysis for the interaction of Instruction × Target Position and for corresponding quadratic trends produced moderate (BF_incl_ = 9.492) and strong (BF_incl_ = 30.454) evidence, respectively, in favor of the effect.

No other effects, including for the corresponding analysis of the error rates, reached significance. The Bayesian analysis for the null effect of the interaction of Instruction × Target Position and for corresponding quadratic trends produced moderate (BF_excl_ = 6.058 and BF_excl_ = 3.017, respectively) evidence against the effect.

See Fig. 2 and Appendix 2 for results.

#### Flanker task

As for the data of Experiments 1 and 2, a 2 × 2 × 2-ANOVA with repeated measures on the factors Congruency (Congruent, Incongruent), Previous Congruency (Congruent, Incongruent) and Instruction (Center, Double) was run on the mean RTs and the error rate of the flanker task. The interaction effect of Congruency and Instruction reached the level of significance *F*(1, 19) = 5.683, *p* = 0.028, *ηp*^*2*^ = 0.23, as flanker interference was reduced following a double instruction (*Congruent* = 674 ms, *Incongruent* = 687 ms), compared with a center instruction (*Congruent* = 692 ms, *Incongruent* = 677 ms). The Bayesian analysis for the effect of Congruency × Instruction, yielded only anecdotal evidence (BF_incl_ = 2.243) in favor of the effect. No other effects, including the corresponding ANOVA of the error rates, reached significance. The Bayesian analysis for the null effect of Congruency × Instruction, yielded only anecdotal evidence against the interaction effect (BF_excl_ = 1.610).

See results in Fig. 3 and Appendix 2.

#### Follow-up interview of adherence to instructions

In a verbal interview following the experiment with open questions (“How did you experience the experiment?”, “Was it possible for you to follow the instructions?”), the following responses differed from the most common response “Quite hard, but I followed the instructions” (15 participants). One participant indicated that the letters became blurred later in the experiment, due to exhaustion. Another participant stated that they did not perform the switch consistently. The interview with a third participant revealed that they did not fully understand what was required for the center/double instruction. Another participant reported following the switching instructions more closely in a later phase than in the earlier phase of the experiment. Data for the verbal interview of Participant 12 were missing; however, the results of the questionnaire following each block (see Sect."Adherence to instructions") indicated low adherence to instructions. Even though the self-assessed adherence data for Participant 17 following each block were missing (see Sect."Adherence to instructions"), the follow-up verbal interview revealed a high degree of understanding of the instructions. After receiving an additional explanation in the practice block, the participant reported that they adherence to the instructions through the experimental session.

### Discussion experiment 3

The procedural modifications implemented in Experiment 3 resulted in a more pronounced center-to-periphery search time gradient in probe trials featuring the center instruction than in probe trials featuring the double instruction. This results suggests, that participants adhered to instructions and responded to the identity of the central letter vs. to the identity of the letter presented twice, narrowing vs. widening their focus of visual attention accordingly. Experiment 3 therefore provided evidence that participants are capable of adjusting their attentional set on a trial-by-trial basis in the absence of any other change made to the task stimuli, responses, stimulus–response mappings, or stimulus-related contingencies. Further support for this interpretation was found in the flanker task performance data. Concerning RTs, the two-way interaction of instruction and congruency took the form of a larger flanker congruency effect in trials featuring the double instruction compared to the center instruction. This pattern aligns with expectations that focusing attention on the central stimulus position occurred to a greater extent in trials featuring the center instruction. In Experiment 1, a similar trend was observed in the error analysis, although it did not reach statistical significance. Unlike Experiment 1, flanker task performance was not overall better with the center compared to the double instruction. Experiment 3 thus did not provide evidence that participants switched to a less efficient strategy (nor that the two strategies differed in efficiency at all).

Demonstrating instruction-dependent search time gradients provided supported for the experimental modification to mitigate the loss of cognitive control (e.g., fatigue) or motivational aspects, which were hypothesized to contribute to the failure of Experiment 2. Because a reduced number of blocks and a manipulation check of self-assessed adherence to instructions were implemented simultaneously, we cannot determine which of these, or both, were necessary to either increase the cognitive control or improve the motivational aspects of the participants.

In contrast to the effect of the trial’s instruction, we observed no effect of the instruction of the preceding (flanker task) trial on the search time gradient in the probe task. This lack of indication of attentional carryover replicates previous findings obtained in situations necessitating switches between tasks associated with different demands of attentional focusing (Kähler et al., 2020; Wendt et al., 2017). In light of the evidence for cue-based adoption of different attentional strategies, this result is consistent with substantial replacement of the previous attentional set during preparation for the upcoming trial. At least two other options appear possible, however. First, it is conceivable that our probe task method lacks sufficient sensitivity to detect attentional carry over. Second, the instructed attentional set of a flanker task trial may be disrupted by a change in context such as switching to the probe task rather than by preparation of the alternative attentional strategy.

## Experiment 4

As mentioned above, previous studies in which an analogous probe task method was used to examine cue-based adjustment of the attentional set under conditions of additional changes of the task (i.e., identifying the central element of a letter string or evaluating a global aspect of the letter string, Kähler et al., 2020; Wendt et al., 2017) yielded no evidence for carryover of the attentional set from the preceding trial to the probe task. To test the hypothesis that (cue-based) preparation for the upcoming task resulted in complete replacement of the previously adopted attentional set, Kähler et al., (2024, Experiment 2B) presented probe task trials without task cues thus preventing task preparation. With this arrangement, carryover of the attentional set adopted on the preceding (flanker task) trial manifested itself as a more pronounced center-to-periphery search time gradient after an Eriksen flanker task trial than after a trial requiring judging whether all letters in the letter string were identical or not. This finding demonstrated that a context change from a flanker task trial to a probe task trial alone was not associated with a degree of disruption of the attentional set that rendered attentional carryover undetectable or that the probe task method lacked sensitivity for detection of attentional carryover for other reasons.

In Experiment 4 of the present study, we applied an analogous method. Specifically, the probe task was presented 500 ms after the response to the preceding trial, that is, at the same time the cue appeared in flanker task trials. Carryover of attentional set adjusted according to the instruction (presented in the previous flanker task trial) should evidence itself as a more pronounced center-to-periphery gradient in search times following a flanker task trial featuring the center instruction than the double instruction.

Participants awareness of our methods for assessing adherence to instructions could have influenced their behavior. To document participants’ possible awareness of this assessment method used to assess their adherence to instructions, we examined their assumptions. Since they were informed during the practice blocks that their responses to the instructions were identical, but we would still measure their adherence to instructions, participants may have wondered about the method more than participants in other experimental settings. These additional data seemed necessary to test if the results of the probe task might be attributable to an awareness of and behavior in line with our measuring method.

### Method

For the general apparatus, stimuli and procedure, see Sect."Method". All changes are outlined below.

#### Participants

The 20 participants had the same background compared to those in Experiment 1–3. The mean age was 21.10 (*SD* = 2.33) with 5 female and 15 male participants.

#### Apparatus, and stimuli

The same Apparatus and Stimuli were used as described in Sect."Apparatus, and stimuli".

#### Procedure

The procedure of the experiment was identical to Experiment 3, with one exception: probe trials were presented without cues, directly 500 ms following the last response.

Additionally, participants filled in a questionnaire following the experimental task, to assess their awareness of our method for measuring their adherence to the (unnecessary) switch between instructions. The first item asked, whether they knew how we measured the shift (No/Yes/I have a guess). The second item instructed participants who answered “Yes” or “I have a guess”, to write down their assumption (open-ended response). The written answer was checked by the experimenter, and if a response was unclear (e.g., “with the digit task”), follow-up questions were asked and documented (see Appendix 1).

### Results

The exclusion and calculation of the experimental data were conducted as described for Experiment 1. For the analysis of probe trials, this resulted in a data loss of 21.14% for RTs and 9.25% for error rates. For the analysis of flanker trials, this resulted in a data loss of 24.89% for RTs and 13.26% for error rates. For the analysis of probe trials excluding data of Participant 11, this resulted in a data loss of 21.06% for RTs and 9.10% for error rates. For the analysis of flanker trials, this resulted in a data loss of 24.96% for RTs and 13.34% for error rates.

#### Participants assumption about the measurement methods

The results and paraphrased responses are presented in Appendix 1. Seven participants indicated that they did not have any assumptions regarding the measurement method. The assumptions made by the 13 participants who did report having one mostly focused on the flanker task or reaction times *in general*. Some mentioned the probe task (*digit task*); however, they speculated about the task in general, rather than about different response rates as a function of the two instructions. One participant’s assumption focused on a specific aspect of the method, as they assumed that focusing on the center would decrease response times to a 7 presented at the bottom. Overall, participants of this experiment were not aware of the probe task measurement.

#### Adherence to instructions

The same procedure as in Experiment 3 was applied (see Sect."Procedure"and"Adherence to instructions"). The mean self-assessed adherence to instructions was 3.63 (*SD* = 0.38). The median of the self-assesses adherence to instruction was at “75–100%” (*N* = 82), followed by 33 blocks with “51–75%”, and only four and one blocks, respectively, “26–50%” and “0–25%”. The range of 2.50 SDs around the mean participants’ mean adherence to instructions was 2.67 to 4.60. The mean adherence to instructions of Participant 11 fell below this threshold. The overall reported adherence to instructions was high (see Tables 2 and 3).

**Table 3:** Self-assessed adherence to instructions by participants (PPs) in Experiment 4, following each experimental block

	PP	1	2	3	4	5	6	7	8	9	10	11	12	13	14	15	16	17	18	19	20
Block																					
1		3	4	3	4	3	4	4	4	4	4	3	4	3	4	4	4	4	4	4	4
2		3	4	4	4	4	4	4	4	3	4	2	3	4	4	4	4	4	4	3	3
3		4	4	3	3	3	4	4	4	4	4	4	3	4	3	3	4	4	4	4	4
4		4	4	2	3	3	4	4	4	3	4	3	3	4	4	4	4	4	4	4	4
5		4	4	3	3	4	3	4	3	4	4	1	4	4	4	3	4	3	4	4	4
6		4	4	3	3	4	4	4	3	3	3	2	3	4	4	2	4	4	4	4	4
mean		3.67	4	3	3.33	3.5	3.83	4	3.67	3.5	3.83	2.5	3.33	3.83	3.83	3.33	4	3.83	4	3.83	3.83

Values were assigned to indicate perceived adherence to instructions as follows:0–25% (1), 26–50% (2), 51–75% (3) and 76–100% (4) of trials in the block

The results for the strategies used following each experimental block demonstrated that most participants primarily applied “both strategies about the same amount” (50.83%). This option aligns with the actual frequencies in the blocks and suggests high compliance. The next most frequent strategy was center (30.50%) followed by double (15.83%). Additionally, 0.83% of data was missing (see Table 4). Overall, this pattern is consistent with the high levels of compliance, as similar usage of both strategies would indicate adherence.

**Table 4:** Self-assessed strategies applied by participants (PPs) in Experiment 4, following each experimental block

Block	1	2	3	4	5	6	7	8	9	10		11	12	13	14	15	16	17	18	19	20
1	3	1	3	3	2	3	3	1	3	3		3	3	NA	1	1	3	3	1	2	3
2	2	3	2	3	3	2	3	1	2	1		1	2	3	2	1	1	1	1	3	1
3	3	2	3	1	1	1	3	1	1	1		3	3	1	2	1	1	3	1	3	2
4	3	3	1	1	3	3	3	1	3	3		1	3	3	3	1	3	3	1	3	3
5	2	3	2	1	3	1	3	3	3	1		1	1	3	3	1	3	2	1	3	2
6	2	2	2	2	3	3	3	3	3	3		1	3	3	3	1	3	3	1	3	3
*N* of Blocks applied both strategies	3	3	2	2	4	3	6	2	4		3	2	4	4	3	0	4	4	0	5	3

Values were assigned to the predominantly applied strategy in the block (1 = “center”, 2 = “double” or 3 = “both to the same amount”)

The lowest compliance was observed in Participant 11 (mean = 2.50), with only two blocks in which they applied both strategies to about the same amount. Next, Participant 3 had a mean compliance of 3.00, with only two blocks in which they applied both strategies to about the same amount. Participants 15 and 4 had a mean compliance of 3.3; however, only two and zero blocks, respectively, in which they applied both strategies to about the same amount. Finally, participants 8 and 18 indicated high mean compliance (3.67 and 4.0, respectively); however, only two and zero blocks, respectively, in which they applied both strategies to about the same amount.

#### Probe task

Because one participant’s mean adherence to instructions was below 2.5 SDs of the sample’s mean adherence to instructions, 2 × 3 ANOVAs with and without that participant were calculated on the mean RTs and the error rates of the probe task with repeated measures on the factors Previous Instruction (Center, Double) and Target Position (Top, Center, Bottom). In the ANOVA of the full sample’s RT, a main effect of Target Position *F*(1.828, 34.723) = 9.842, *p* = 0.001, *ηp*^*2*^ = 0.341 reached significance, as responses were faster in the central (833 ms) compared to the peripheral positions (*Top* = 860 ms, *Bottom* = 879 ms). The interaction of Previous Instruction × Target Position reached a trend F(1.887, 35.857) = 2.974, p = 0.067, *ηp*^*2*^ = 0.135 which did not reach significance in the planned comparisons of the quadratic trends *F*(1, 19) = 3.051, *p* = 0.097, *ηp*^*2*^ = 0.138, demonstrating only a numerically more pronounced center-to-periphery gradient after the previous center instruction (*Top* = 869 ms, *Center* = 820 ms, *Bottom* = 870 ms) compared with that of the previous double instruction (*Top* = 852 ms, *Center* = 846 ms, *Bottom* = 888 ms). The Bayesian analysis for the interaction of Previous Instruction × Target Position and for corresponding quadratic trends including Participant 11, yielded only anecdotal evidence (BF_incl_ = 2.406 and BF_incl_ = 1.343, respectively) in favor of the effect.

In contrast, in the equivalent ANOVAs without Participant 11, the interaction of interest, namely, Previous Instruction × Target Position *F*(1.847, 33.247) = 3.426, *p* = 0.048, *ηp*^*2*^ = 0.16 reached significance and was confirmed by the corresponding planned comparison *F*(1, 18) = 6.177, *p* = 0.023, *ηp*^*2*^ = 0.255, demonstrating a more pronounced center-to-periphery gradient after a previous center instruction (*Top* = 871 ms, *Center* = 819 ms, *Bottom* = 876 ms) compared with that of a double instruction (*Top* = 856 ms, *Center* = 850 ms, *Bottom* = 888 ms). The Bayesian analysis for the interaction of Previous Instruction × Target Position and for corresponding quadratic trends without Participant 11, produced moderate evidence (BF_incl_ = 3.132 and BF_incl_ = 4.599, respectively) in favor of the effect.

For the error rates, the main effect of Previous Instruction reached the level of significance *F*(1, 19) = 6.209, *p* = 0.022, *ηp*^*2*^ = 0.246, due to higher error rates following the previous center instruction (5.60%) compared to trials following the double instruction (3.40%). The Bayesian analysis for the null effect of the interaction of Previous Instruction × Target Position and for corresponding quadratic trends including Participant 11 produced moderate (BF_excl_ = 5.947) and anecdotal (BF_excl_ = 2.816) evidence, respectively, against the effect.

No change in the pattern of results was found in the analysis of error rates excluding data of Participant 11. The Bayesian analysis for the null effect of the interaction of Previous Instruction × Target Position and for corresponding quadratic trends without Participant 11 produced moderate (BF_excl_ = 5.739) and anecdotal (BF_excl_ = 2.646) evidence, respectively, against the effect.

Full results are presented in Appendix 2 and Fig. 2.

#### Flanker task

**Fig. 4:**
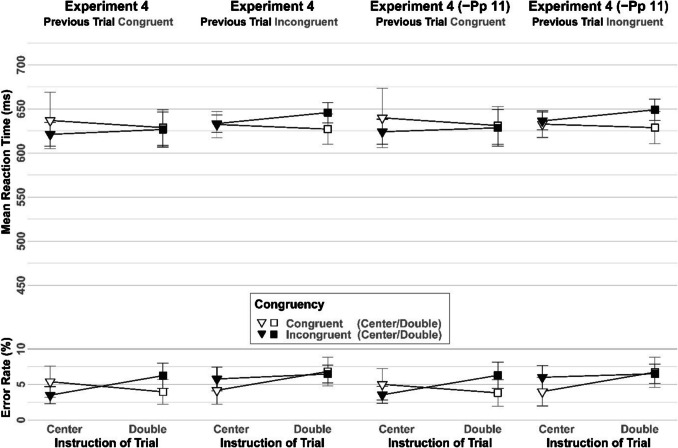
Mean reaction times and error rates in the flanker-task of Experiment 4 as a function of Instruction (Center, Double), Congruency (Congruent, Incongruent) and Previous Congruency (Congruent, Incongruent). Error bars reflect the 95% within-confidence Intervals.– Pp11 = Participant 11 excluded

The 2 × 2 × 2-ANOVAs with repeated measures on the factors Congruency (Congruent, Incongruent), Previous Congruency (Congruent, Incongruent), and Instruction (Center, Double) were run on the mean RTs and error rates for the sample, both with and without Participant 11. The interaction of Congruency × Previous Congruency was marginally significant in the complete sample *F*(1, 19) = 3.801, *p* = 0.066, *ηp*^*2*^ = 0.167 and reached significance in the analysis excluding data of Participant 11 *F*(1, 18) = 4.609, *p* = 0.046, *ηp*^*2*^ = 0.204. As can be seen in Fig. 4, RTs were slower when the congruency level repeated compared to when it switched from the previous trial. The Bayesian analysis for the effect of Congruency × Previous Congruency excluding data of Participant 11, yielded only anecdotal evidence in favor of the interaction effect (BF_incl_ = 1.179).

The Bayesian analysis for the null effect of Congruency × Instruction including Participant 11, yielded anecdotal evidence against the interaction effect (BF_excl_ = 1.487). Similarly, the Bayesian analysis for the null effect of Congruency × Instruction excluding data of Participant 11, yielded only anecdotal evidence against the interaction effect (BF_excl_ = 1.759).

The analogous analysis of error rates yielded a significant 3-way interaction of Congruency × Previous Congruency × Instruction both with and without the data of the Participant 11 *F*(1, 19) = 6.40, *p* = 0.02, *ηp*^*2*^ = 0.252 and excluding Participant 11 *F*(1, 18) = 5.894, *p* = 0.026, *ηp*^*2*^ = 0.247. The Bayesian analysis for the effect of Congruency × Previous Congruency × Instruction, including Participant 11, yielded very strong evidence (BF_incl_ = 46.624) in favor of the interaction effect. Similarly, the Bayesian analysis for the effect of Congruency × Previous Congruency × Instruction, without Participant 11, yielded very strong evidence (BF_incl_ = 36.107) in favor of the interaction effect. As displayed in Fig. 4, the 3-way interaction can be described as a congruency level repetition disadvantage in trials with the center instruction and a (less pronounced) congruency level repetition advantage in trials with the double instruction.

For full results see Appendix 2 and Fig. 4.

### Discussion experiment 4

Experiment 4 yielded preliminary evidence of carryover of attentional sets adopted on the basis of instructional cues, provided sufficient adherence to the instructions. Specifically, after exclusion of the data of one participant who reported extremely low adherence to instruction compared to the mean adherence to instruction of the sample, the center-to-periphery gradient of search times was more pronounced after a flanker task trial featuring the center instruction than after a flanker task trial featuring the double instruction. Firstly, this interpretation implies sufficient sensitivity of the probe task to detect attentional carryover, questions the idea that a contextual change between the two tasks results in a fatal disruption of the previously adopted attentional set. Secondly, it also provides further support for the notion that participants adopted the attentional strategy indicated by the cue in the flanker task trials (because there could be no carryover to the subsequent trial otherwise). The results of Experiment 4 should be considered with caution, however, as exclusion of the data of one participant was not based on criteria defined a priori. Furthermore, while the results of the flanker task did not yield additional evidence for instruction-based adjustment of the attentional set (i.e., no larger congruency effect in trials featuring the double instruction than in trials featuring the center instruction) they even displayed a surprising disadvantage when repeating the congruency level in RTs and, for trials associated with the center instruction, also in error rates. We have no explanation for this finding.

## General discussion

Research on attention primarily relies on assessing performance measures in tasks for which participants are instructed to select a response according to a set of instructed stimulus–response transformation rules. In this study, we presented evidence for the facultative adoption of attentional sets resulting from instructed cognitive strategies in otherwise identical task and stimulus environments. Using a probe task sensitive to attentional strategy, we demonstrated shifts in attentional sets under otherwise constant contextual conditions when the instruction was switched once during the experiment (Experiment 1) and when the instruction was switched on a trial-to-trial basis (Experiment 3). Finally, we provided preliminary evidence for carryover of attentional sets thus adopted to an unrelated visual search task (Experiment 4).

Accuracy and/or speed of responding is usually compared between conditions associated with contextual variations assumed to affect particular processes within the chain of stimulus–response processing. Such effects should reveal themselves as performance differences between conditions. Although the occurrence of certain abstract cognitive operations can be inferred from an above-chance level of accuracy (e.g., some form of extraction of stimulus information that allows discriminating among choice alternatives), the precise mental operations or strategies involved in solving the task typically remain unknown. Systematic variations in task strategies have been suggested in some particular contexts. For instance, it has been conjectured that the cognitive processes involved in solving the same task might change during practice (e.g., Logan, 1988) or that different populations (e.g., females and males) tend to rely on different types of information for solving the same task (e.g., Raabe et al., 2006).

It seems reasonable to assume that typical laboratory tasks used in attention research might be accomplished (i.e., a satisfactory outcome may be achieved) in multiple ways concerning the precise mental processes involved. Although standard performance measures might not be capable of detecting different strategies among individuals or experimental conditions, some clarity may be gained by instructing participants explicitly to apply a particular strategy to solve a given task and comparing task performance for different instructions. In a programmatic article, Leber & Irons (2019) pleaded for using a large variety of experimental manipulations and assessments to investigate “attentional strategy” (defined as a “mental plan, or policy, guiding how individuals prioritize and select sensory information”, p. 274). One of the methods they presented in their “research toolbox” pertained to manipulating instructions and assessing their effects on the processing of otherwise identical tasks. Although observing performance differences under such circumstances provides evidence that instructions did influence task processing, it does not guarantee that *the instructed strategies* were indeed adopted. To achieve the latter kind of evidence performance patterns indicative of the applied strategy (“markers”) are necessary. Complementing the manipulation of instructions with the probe task methodology we applied in the experiments of the current article aimed at yielding such evidence.

In the introduction, we pointed out that experimental evidence of shifts of the attentional set in the absence of a shift of response demands or stimulus-related contingencies is sparse—contrasting with introspective intuitions of our capability of shifting the attentional set by purely endogenous means. Although the experiments of the current study provided such evidence by keeping response demands and stimulus-related contingencies constant, it is still noteworthy that this was obtained under somewhat special circumstances. These circumstances allowed task completion (i.e., executing the correct response) by application of two discernibly different mental operations (i.e., identifying the letter presented at the central location and identifying the letter presented twice, respectively), presumably associated with attentional sets of different focus width (instead of directly instructing the participant to “focus your attention on the central position” and “widen your attention to the entire string”). It seems likely that shifting of the attentional set occurred as a by-product of switching between these cognitive operations. This would differ strikingly from the type of “direct” manipulation of attentional weights given to target- and distractor-related information—unaccompanied by other changes in model parameters—assumed to occur in response to certain critical events in information processing such as the detection of response conflict (e.g., Botvinick et al., 2001). Our results thus leave open to what extent purely intention-based adjustments of the attentional set can be accomplished in the absence of a discernable possibility of applying distinct cognitive operations associated with different attentional sets.

Further limitations of our study deserve discussion. First, although our method ensured equivalent stimulus presentation conditions and response requirements following cues instructing responding to the centrally presented letter vs. to the letter presented twice, the cues themselves differed in terms of physical appearance and, given that participants adhered to instructions at least to some extent, also concerning “selection history”. Even though it seems unlikely to us that the particular shape of the words used evoked a difference in the distribution of attentional weights across the three stimulus locations that resulted in a different search time gradient, assuming that associations are formed between contextual stimulus features (such as a cue presented in advance of the imperative stimulus) and control settings adopted at the time (such as the set of visual attention), implementation of a narrower or wider focus of attention may have been supported more and more strongly by associations formed during previous application during the course of the experimental session (see, e.g., Gade & Koch, 2007, for evidence of the formation of practice-dependent associations between cues and task-sets in task cueing protocols). This caveat may be addressed in future studies by instructing different attentional strategies in regular sequences rather than by presentation of explicit cues, or by asking participants to choose a strategy voluntarily on each trial.

Second, unlike experiments which investigated adjustment of overt attention by analyzing eye movements during task preparation when target stimuli were presented at different (task-specific) locations on the screen (e.g., Longman et al., 2014), obtaining differentially steep search time gradients in probe task trials following cues instructing different processing strategies does not necessarily imply that the attentional adjustment took place during preparation. It is also conceivable that the adjustment occurred only after presentation of the imperative stimulus (i.e., after encoding of the stimulus of a flanker task trial or a probe task trial). As our primary measure of the attentional set was the search time gradient of the probe task—and flanker interference was hardly ever lower with the instruction to respond to the central letter than with the instruction to respond to the letter presented twice—it is conceivable that attentional adjustment was largely confined to conditions in which the task stimulus differed markedly from the stimulus presented in the preceding trial. In other words, although stimulus conditions following the two cues were identical, we cannot not dismiss the possibility that a change in stimulus conditions is needed for the shift of the attentional set to occur.

Finally, even the evidence obtained in the probe task trials is somewhat weakened by the failure of observing a difference in the search time gradient in Experiment 2 and by achieving statistical significance in Experiment 4 only after the data of one participant were eliminated from the analyses. Although we lack compelling evidence that this equivocality can be ascribed to unfavorable motivational conditions, exploratory re-analyses based on the data of sub-groups of participants of Experiment 3 and Experiment 4 reporting high levels of adherence to instructions are consistent with this assumption. These analyses are reported in Appendix 3. Independently of the precise mechanisms by which the adjustment of the attentional set is brought about and of the necessary preconditions, our approach enables a fresh way to investigate participants’ motivation and ability to adhere to instructions given by the experimenter. Manipulations of factors such as the relative difficulty of task strategies or of participants’ presumption concerning the experimenter’s capability of identifying the strategy used on a given trial appear to be interesting candidates for further research.

## Appendix 1

see Table 5

**Table 5:** Follow-up questionnaire of experiment 4 as a manipulation check for participants’ potential assumptions about the measuring method

		Pp. No	Written answer of Participants (translated from German)	Transcribed verbal responses to the experimenter’s question for a more detailed description of their hypothesis (paraphrased documenter from the experimenter and translated from German)
Do you know how we controlled if you switched the tasks?	No	4, 5, 9, 13, 16, 19, 20	-	-
	Yes	1	With the ratio between Central and Double	Of the flanker task
	I have a guess			
		1	With the digits	No further assumptions
		2	With shorter RTs when the instruction is “central”	Overall faster
		3	With the ratio of RT central-double	In the flanker task
		6	With the digits and sequence after the letters	Sequence of Instructions: if wrong answer, more problems if only focused on center. Worse/slower in digits overall
		7	With eye-tracking, or with RTs	For the RTs no further assumptions
		8	With the digit task, shift of 3 and 7 up or down	If focus on center, then 7 slower on the bottom
		10	Digits manipulated me, I probably expected rather a double task following a digit on top or bottom	
		11	With RT, that differed in case of “Central” or “double”	Longer in double instruction (flanker task) Hypothesized, that the digit task was used to test if participants are awake
		12	With fluctuation in RT	No further assumptions
		14	With sequence of digits	If double task, then digit at the bottom
		15	Shorter RTs despite task switch	In the flanker task
		17	Shortly after, the digits followed in opposite direction. Maybe with the RTs of focusing it is measurable	In case of a digit on the top position, a following response in flanker task was slower
		18	Time was measured	No further assumptions, just measured time

Note that for participants the term “task” was used to refer to the two instructions

## Appendix 2

see Tables 6, 7, 8, 9, 10, 11, 12, 13, 14, 15, 16, 17, 18, 19, 20, 21, 22, 23, 24, 25

**Table 6:** ANOVA of the probe task RTs of experiment 1 with repeated measures on the factors Instruction (Center, Double), target position (Top, Center, Bottom) and the between-factor start-condition (Center, Double)

*Predictor*	*df*_*Num*_	*df*_*Den*_	*MSE*	*F*	*η*_*p*_^*2*^	*p*	
Start Condition (SC)	1	18	62627.85	0.20	0.011	0.658	
Instruction (I)	1	18	4038.91	3.64	0.168	0.073	+
SC × I	1	18	4038.91	6.25	0.258	0.022	*
Target Position (TP)	1.97	35.46	1231.79	28.61	0.614	<.001	***
SC × TP	1.97	35.46	1231.79	1.02	0.054	0.370	
I × TP	1.97	35.37	735.27	7.68	0.299	0.002	**
SC × I × TP	1.97	35.37	735.27	0.05	0.003	0.954	

**Table 7:** ANOVA of the probe task error rates of experiment 1 with repeated measures on the factors Instruction (Center, Double), Target Position (Top, Center, Bottom) and the between-factor Start-Condition (Center, Double)

*Predictor*	*df*_*Num*_	*df*_*Den*_	*MSE*	*F*	*η*_*p*_^*2*^	*p*	
Start Condition (SC)	1	18	226.69	0.15	0.008	0.702	
Instruction (I)	1	18	19.88	0.05	0.003	0.824	
SC × I	1	18	19.88	1.36	0.07	0.259	
Target Position (TP)	1.74	31.39	22.76	11.6	0.392	<.001	***
SC × TP	1.74	31.39	22.76	0.04	0.002	0.946	
I × TP	1.99	35.78	15.48	6.22	0.257	0.005	**
SC × I × TP	1.99	35.78	15.48	2.42	0.119	0.103	

**Table 8:** ANOVA of the flanker task RTs of experiment 1 with repeated measures on the factors Congruency (Congruent, Incongruent), Previous Congruency (Congruent, Incongruent) and Instruction (Center, Double)

*Predictor*	*df*_*Num*_	*df*_*Den*_	*MSE*	*F*	*η*_*p*_^*2*^	*p*	
Congruency (C)	1	19	113.80	13.01	0.406	0.002	**
Previous Congruency (PrC)	1	19	195.39	0.61	0.031	0.444	
Instruction (I)	1	19	2066.86	12.84	0.403	0.002	**
C × PrC	1	19	102.84	1.32	0.065	0.265	
C × I	1	19	69.40	0.46	0.024	0.505	
PrC × I	1	19	322.61	0.36	0.019	0.556	
C × PC × I	1	19	126.07	0.35	0.018	0.563	

**Table 9:** ANOVA of the flanker task error rates of experiment 1 with repeated measures on the factors Congruency (Congruent, Incongruent), Previous Congruency (Congruent, Incongruent) and Instruction (Center, Double)

*Predictor*	*df*_*Num*_	*df*_*Den*_	*MSE*	*F*	*η*_*p*_^*2*^	*p*	
Congruency (C)	1	19	17.21	1.85	0.089	0.190	
Previous Congruency (PrC)	1	19	4.34	1.30	0.064	0.268	
Instruction (I)	1	19	10.56	2.20	0.104	0.154	
C × PrC	1	19	4.23	<.001	<.001	0.982	
C × I	1	19	10.10	3.18	0.143	0.091	+
PrC × I	1	19	7.17	0.001	<.001	0.978	
C × PrC × I	1	19	14.48	2.01	0.096	0.172	

**Table 10:** ANOVA of the probe task RTs of experiment 2 with repeated measures on the factors Instruction (Center, Double), Previous Instruction (Center, Double) and Target Position (Top, Center, Bottom)

*Predictor*	*df*_*Num*_	*df*_*Den*_	*MSE*	*F*	*η*_*p*_^*2*^	*p*	
Instruction (I)	1	19	955.44	0.01	<.001	0.919	
Previous Instruction (PrI)	1	19	829.36	0.55	0.028	0.469	
Target Position (TP)	1.92	36.39	1857.89	44.5	0.701	<.001	***
I × PrI	1	19	1699.35	0.27	0.014	0.608	
I × TP	1.62	30.85	2107.56	1.06	0.053	0.347	
PrI × TP	1.59	30.22	1525.74	0.91	0.046	0.391	
I × PrI × TP	1.96	37.16	714.63	0.69	0.035	0.505	

**Table 11:** ANOVA of the probe task error rates of experiment 2 with repeated measures on the factors Instruction (Center, Double), Previous Instruction (Center, Double) and Target Position (Top, Center, Bottom)

*Predictor*	*df*_*Num*_	*df*_*Den*_	*MSE*	*F*	*η*_*p*_^*2*^	*p*	
Instruction (I)	1	19	44.39	0.27	0.014	0.606	
Previous Instruction (PrI)	1	19	17.81	1.66	0.08	0.213	
Target Position (TP)	1.78	33.83	28.48	8.49	0.309	0.001	**
I × PrI	1	19	34.85	0.03	0.002	0.858	
I × TP	1.62	30.87	17.66	0.40	0.021	0.631	
PrI × TP	1.87	35.53	18.62	1.28	0.063	0.290	
I × PrI × TP	1.76	33.53	37.79	0.49	0.025	0.596	

**Table 12:** ANOVA of the flanker task RTs of experiment 2 with repeated measures on the factors Congruency (Congruent, Incongruent), Previous Congruency (Congruent, Incongruent) and Instruction (Center, Double)

*Predictor*	*df*_*Num*_	*df*_*Den*_	*MSE*	*F*	*η*_*p*_^*2*^	*p*	
Congruency (C)	1	19	204.61	3.16	0.143	0.091	+
Previous Congruency (PrC)	1	19	102.42	0.06	0.003	0.816	
Instruction (I)	1	19	504.89	0.39	0.02	0.541	
C × PrC	1	19	102.89	0.86	0.043	0.366	
C × I	1	19	201.48	0.63	0.032	0.436	
PrC × I	1	19	196.85	0.08	0.004	0.775	
C × PrC × I	1	19	357.39	0.7	0.035	0.415	

**Table 13:** ANOVA of the flanker task error rates of experiment 2 with repeated measures on the factors Congruency (Congruent, Incongruent), Previous Congruency (Congruent, Incongruent) and Instruction (Center, Double)

*Predictor*	*df*_*Num*_	*df*_*Den*_	*MSE*	*F*	*η*_*p*_^*2*^	*p*	
Congruency (C)	1	19	10.18	4.53	0.192	0.047	*
Previous Congruency (PrC)	1	19	7.11	2.80	0.129	0.110	
Instruction (I)	1	19	6.34	1.52	0.074	0.232	
C × PrC	1	19	4.26	0.22	0.012	0.643	
C × I	1	19	5.34	1.13	0.056	0.301	
PrC × I	1	19	14.13	0.28	0.014	0.606	
C × PrC × I	1	19	10.72	1.29	0.064	0.270	

**Table 14:** ANOVA of the probe task RTs of experiment 3 with repeated measures on the factors Instruction (Center, Double), Previous Instruction (Center, Double) and Target Position (Top, Center, Bottom)

*Predictor*	*df*_*Num*_	*df*_*Den*_	*MSE*	*F*	*η*_*p*_^*2*^	*p*	
Instruction (I)	1	19	2925.69	1.03	0.051	0.324	
Previous Instruction (PrI)	1	19	1675.96	0.01	<.001	0.940	
Target Position (TP)	1.68	31.92	5598.81	8.83	0.317	0.002	**
I × PrI	1	19	4579.74	0.04	0.002	0.835	
I × TP	1.90	36.13	3711.28	6.46	0.254	0.005	**
PrI × TP	1.99	37.9	2377.43	0.44	0.023	0.648	
I × PrI × TP	1.79	33.97	4282.63	1.08	0.054	0.344	

**Table 15:** ANOVA of the probe task error rates of experiment 3 with repeated measures on the factors Instruction (Center, Double), Previous Instruction (Center, Double) and Target Position (Top, Center, Bottom)

*Predictor*	*df*_*Num*_	*df*_*Den*_	*MSE*	*F*	*η*_*p*_^*2*^	*p*
Instruction (I)	1	19	30.22	0.34	0.018	0.567
Previous Instruction (PrI)	1	19	58.03	0.10	0.005	0.758
Target Position (TP)	1.87	35.53	41.68	1.57	0.076	0.223
I × PrI	1	19	72.92	0.05	0.003	0.828
I × TP	1.95	37.08	66.69	0.01	<.001	0.994
PrI × TP	1.58	29.99	69.65	1.54	0.075	0.232
I × PrI × TP	1.83	34.79	46.46	0.44	0.023	0.629

**Table 16:** ANOVA of the flanker task RTs of experiment 3 with repeated measures on the factors Congruency (Congruent, Incongruent), Previous Congruency (Congruent, Incongruent) and Instruction (Center, Double)

*Predictor*	*df*_*Num*_	*df*_*Den*_	*MSE*	*F*	*η*_*p*_^*2*^	*p*	
Congruency (C)	1	19	782.8	0.04	0.002	0.840	
Previous Congruency (PrC)	1	19	1182.5	2.51	0.117	0.129	
Instruction (I)	1	19	2154.09	0.27	0.014	0.608	
C × PrC	1	19	950.03	0.05	0.003	0.825	
C × I	1	19	1317.05	5.68	0.230	0.028	*
PrC × I	1	19	1198.36	0.02	0.001	0.877	
C × PrC × I	1	19	1322.03	2.59	0.12	0.124	

**Table 17:** ANOVA of the flanker task error rates of experiment 3 with repeated measures on the factors Congruency (Congruent, Incongruent), Previous Congruency (Congruent, Incongruent) and Instruction (Center, Double)

*Predictor*	*df*_*Num*_	*df*_*Den*_	*MSE*	*F*	*η*_*p*_^*2*^	*p*
Congruency (C)	1	19	10.68	2.37	0.111	0.140
Previous Congruency (PrC)	1	19	7.50	0.05	0.002	0.834
Instruction (I)	1	19	18.00	1.53	0.075	0.231
C × PrC	1	19	8.56	1.02	0.051	0.325
C × I	1	19	8.73	2.45	0.114	0.134
PrC × I	1	19	13.98	0.97	0.049	0.337
C × PrC × I	1	19	17.81	0.14	0.007	0.710

**Table 18:** ANOVA of the probe task RTs of experiment 4, including participant 11, with repeated measures on the factors Previous Instruction (Center, Double) and Target Position (Top, Center, Bottom)

*Predictor*	*df*_*Num*_	*df*_*Den*_	*MSE*	*F*	*η*_*p*_^*2*^	*p*	
Previous Instruction (PrI)	1	19	1211.96	2.01	0.095	0.173	
Target Position (TP)	1.83	34.72	2321.51	9.84	0.341	<.001	***
PrI × TP	1.89	35.86	1836.12	2.97	0.135	0.067	+

**Table 19:** ANOVA of the probe task error rates of experiment 4, including participant 11, with repeated measures on the factors Previous Instruction (Center, Double) and Target Position (Top, Center, Bottom)

*Predictor*	*df*_*Num*_	*df*_*Den*_	*MSE*	*F*	*η*_*p*_^*2*^	*p*	
Previous Instruction (PrI)	1	19	23.26	6.21	0.246	0.022	*
Target Position (TP)	1.66	31.45	25.48	0.35	0.018	0.665	
PrI × TP	1.77	33.69	24.39	0.25	0.013	0.758	

**Table 20:** ANOVA of the probe task RTs of experiment 4, participant 11 excluded (see Sect."Probe task"), with repeated measures on the factors Previous Instruction (Center, Double) and Target Position (Top, Center, Bottom)

*Predictor*	*df*_*Num*_	*df*_*Den*_	*MSE*	*F*	*η*_*p*_^*2*^	*p*	
Previous Instruction (PrI)	1	18	1263.91	2.12	0.105	0.163	
Target Position (TP)	1.82	32.79	2389.89	10.03	0.358	<.001	***
PrI × TP	1.85	33.25	1622.85	3.43	0.16	0.048	*

**Table 21:** ANOVA of the probe task error rates of experiment 4, participant 11 excluded (see Sect."Probe task"), with repeated measures on the factors Previous Instruction (Center, Double) and Target Position (Top, Center, Bottom)

*Predictor*	*df*_*Num*_	*df*_*Den*_	*MSE*	*F*	*η*_*p*_^*2*^	*p*	
Previous Instruction (PrI)	1	18	23.96	5.03	0.219	0.038	*
Target Position (TP)	1.65	29.78	26.89	0.34	0.018	0.677	
PrI × TP	1.77	31.92	25.75	0.23	0.013	0.770	

**Table 22:** ANOVA of the flanker task RTs of experiment 4, including participant 11 (see Sect."Probe task"), with repeated measures on the factors Congruency (Congruent, Incongruent), Previous Congruency (Congruent, Incongruent) and Instruction (Center, Double)

*Predictor*	*df*_*Num*_	*df*_*Den*_	*MSE*	*F*	*η*_*p*_^*2*^	*p*	
Congruency (C)	1	19	1552.31	0.01	< 0.001	0.942	
Previous Congruency (PrC)	1	19	1371.50	1.15	0.057	0.297	
Instruction (I)	1	19	2845.65	0.02	0.001	0.889	
C × PrC	1	19	929.20	3.80	0.167	0.066	+
C × I	1	19	996.50	2.46	0.115	0.133	
PrC × I	1	19	1948.78	0.12	0.006	0.732	
C × PrC × I	1	19	1164.54	0.04	0.002	0.850	

**Table 23:** ANOVA of the flanker task error rates of experiment 4, including participant 11 (see Sect."Probe task"), with repeated measures on the factors Congruency (Congruent, Incongruent), Previous Congruency (Congruent, Incongruent) and Instruction (Center, Double)

*Predictor*	*df*_*Num*_	*df*_*Den*_	*MSE*	*F*	*η*_*p*_^*2*^	*p*	
Congruency (C)	1	19	16.72	0.41	0.021	0.532	
Previous Congruency (PrC)	1	19	14.50	2.98	0.136	0.100	
Instruction (I)	1	19	14.35	3.77	0.166	0.067	+
C × PrC	1	19	13.56	0.13	0.007	0.719	
C × I	1	19	9.82	1.26	0.062	0.276	
PrC × I	1	19	15.92	0.65	0.033	0.429	
C × PrC × I	1	19	14.59	6.40	0.252	0.020	*

**Table 24:** ANOVA of the flanker task RTs of experiment 4, participant 11 excluded (see Sect."Probe task"), with repeated measures on the factors Congruency (Congruent, Incongruent), Previous Congruency (Congruent, Incongruent) and Instruction (Center, Double)

*Predictor*	*df*_*Num*_	*df*_*Den*_	*MSE*	*F*	*η*_*p*_^*2*^	*p*	
Congruency (C)	1	18	1602.48	0.05	0.003	0.833	
Previous Congruency (PrC)	1	18	1438.56	0.89	0.047	0.357	
Instruction (I)	1	18	3003.26	0.01	<.001	0.904	
C × PrC	1	18	922.10	4.61	0.204	0.046	*
C × I	1	18	1045.71	2.01	0.101	0.173	
PrC × I	1	18	2030.4	0.19	0.011	0.664	
C × PrC × I	1	18	1228.08	0.02	0.001	0.881	

**Table 25:** ANOVA of the flanker task error rates of experiment 4, participant 11 excluded (see Sect."Probe task"), with repeated measures on the factors Congruency (Congruent, Incongruent), Previous Congruency (Congruent, Incongruent) and Instruction (Center, Double)

*Predictor*	*df*_*Num*_	*df*_*Den*_	*MSE*	*F*	*η*_*p*_^*2*^	*p*	
Congruency (C)	1	18	14.18	1.31	0.068	0.268	
Previous Congruency (PrC)	1	18	14.83	3.36	0.157	0.083	+
Instruction (I)	1	18	15.11	3.58	0.166	0.075	+
C × PrC	1	18	14.31	0.10	0.006	0.750	
C × I	1	18	9.63	0.71	0.038	0.411	
PrC × I	1	18	16.49	0.41	0.022	0.528	
C × PrC × I	1	18	15.39	5.89	0.247	0.026	*

## Appendix 3 Post hoc analysis of the effects of adherence to instruction

As the two instructions for different task processing strategies did not differ concerning the required responses in flanker and probe task trials, using an error-rate threshold to exclude “bad data” was impossible in this kind of experimental setting. We did not define a priori criteria for exclusion of data from individual participants based on self-reported levels of compliance and applied strategy assessed following each block of Experiments 3 and 4. We excluded one participant of Experiment 4 from the analysis because the mean compliance was below 2.5 SDs the sample’s mean. In a more fine-grained approach for excluding data from non-adhering participants one might combine the two sources of information about the adherence to instructions from each participant following the six experimental blocks. Non-adhering participants were selected based on either reporting low mean compliance over the six blocks (below 3) or reporting to have applied both strategies equally frequently in less than half of the trial blocks (which would be expected if they adhere to the equally frequently presented instructions). The experimental data of non-adhering participants is hypothesized to impair the results of instruction-dependent adoption of attentional sets of the experiment (i.e. gradient of search times in the probe task). Searching for a possible predictive value of the two additional measures of adherence to instruction following each block for the gradients of search time in the probe task could be utilized to define cut-off criteria for the exclusion of non-adhering participants based on their self-assessed adherence to instruction in upcoming research.

We repeated the critical ANOVAs conducted for Experiment 3 and Experiment 4, excluding data from participants likely to have displayed low levels of adherence to instructions (see Sect."Probe task"and"Probe task"). The error analysis did not result in significant results for the interaction of interest (i.e. Instruction × Target Position for Experiment 3 and Previous Instruction × Target Position for Experiment 4) in the ANOVA and corresponding Bayes Factor. Therefore, we report only results for reaction times. All analyses reported in this appendix were exploratory. Effects were not tested for statistical significance.

To begin with Experiment 3, Participant 01 had the lowest compliance, on average, followed by Participants 09 and 12 (on average, 2.00, 2.17, and 2.33, respectively; see Table 1). In addition, all three participants indicated a small number of blocks in which they followed the instructions equally frequently (overall in two, zero, and one block, respectively for Participants 01, 09, and 12). The corresponding ANOVA without those three participants'data resulted in a larger effect size (compared to the ANOVA of the complete sample) of the interaction Target Position × Instruction* F*(2, 32) = 8.124, *p* = 0.001, *ηp*^*2*^ = 0.337 and for the planned comparison of quadratic trends *F*(1, 16) = 20.775, *p* < 0.001, *ηp*^*2*^ = 0.565. The Bayesian analysis for the interaction of Target Position × Instruction and corresponding quadratic trends produced strong (BF_incl_ = 19.894) and very strong (BF_incl_ = 76.523) evidence, respectively, in favor of the interaction effect. See Appendix Table 26 for an overview of the analysis.

Participant 06 reported similarly low compliance as the participants mentioned before (mean = 2.00); however, they reported performing both strategies equally frequently in all six blocks. Participant 08 had an, on average, larger compliance of 3.17, however, reported only one block of following both instructions at about the same time. Eliminating these two participants'data, in addition to the other three, resulted in an even larger effect size for the interaction of Target Position × Instruction *F*(2, 28) = 8.029, *p* = 0.002, *ηp*^*2*^ = 0.364 and the planned comparison of quadratic trends *F*(1, 14) = 20.982, *p* < 0.001, *ηp*^*2*^ = 0.600. The Bayesian analysis for the interaction of Target Position × Instruction and corresponding quadratic trends produced strong (BF_incl_ = 19.715) and very strong (BF_incl_ = 73.797) evidence, respectively, in favor of the interaction effect.

Finally, additional elimination of the data of Participants 05 and 04 (which both indicated mean compliance of 4.00, with either two or zero blocks, respectively, following both strategies equally frequently) increased the effect sizes even further (interaction of Target Position × Instruction *F*(2, 24) = 8.943, *p* = 0.001, *ηp*^*2*^ = 0.427, planned comparison of quadratic trends *F*(1, 12) = 20.353, *p* < 0.001, *ηp*^*2*^ = 0.629). The Bayesian analysis for the interaction of Target Position × Instruction and corresponding quadratic trends produced strong (BF_incl_ = 23.307) and very strong (BF_incl_ = 76.493) evidence, respectively, in favor of the interaction effect.

The Bayesian inclusion factor for the interaction and corresponding quadratic trends indicated that the effects did not improve substantially following the additional exclusion of participants’ data, beyond the initial removal of the three participants who reported very low mean compliance. The Bayes Factors doubled to a strong level for the interaction (16–23) and a very strong level (between 73 and 77) for the quadratic trends, following the first and consecutive exclusion of data. This reanalysis supports the effect of instruction-based adoption of attentional sets, the benefit of excluding low-compliance participants, and the validity of the self-assessments for evaluating participant’s experimental data. However, excluding participants 04 and 05 data did not improve the effect size and Bayes factor. It might be beneficial to assess, instead or in addition, the proportions in which participants performed the indicated preferred strategy. If the two instructions are presented equally frequently, a proportion of 40/60, when following the actual instruction, might be enough to demonstrate the attentional adjustment (compared to a proportion of 90/10).

A summary of these analyses is presented in Appendix Table 26.

**Table 26:** Summary of the re-analysis of the interaction of interest (Target Position × Instruction) with the corresponding planned comparison of quadratic trends of Experiment 3. Presented are the effect sizes (ηp) of the effects (for full results see the text of Appendix 3). The rows indicate the participants whose data were eliminated from the analysis (successively more from top to bottom), as well as the criteria for the data-elimination; the self-assessed compliance and strategy values

Participants’ data excluded in this step	*N*	ηp^2^ of target Position × Instruction *(ANOVA/, Planned comparison of quadratic trends)*	BF_incl_ Target Position × Instruction *(ANOVA/, Planned comparison of quadratic trends)*	Mean compliance over six blocks	Blocks of applying both strategies
None	20	(*ηp*^*2*^ = 0.254/*ηp*^*2*^ = 0.430)	(9.492/30.454)	*Sample Mean* = 3.44	*Sample Mean* = 3.35
Wo 01				2.00	2
and 09				2.17	0
and 12	17	*ηp*^*2*^ = 0.337/*ηp*^*2*^ = 0.565)	(19.894/76.523)	2.33	1
Wo 06				2.00	6
and 08	15	(*ηp*^*2*^ = 0.364/*ηp*^*2*^ = 0.600)	(19.715/73.797)	3.17	1
Wo 05				4.00	2
and 04	14	(*ηp*^*2*^ = 0.427/*ηp*^*2*^ = 0.629)	(23.307/76.493)	4.00	0

*wo* without, *N* sample size, *ηp*^*2*^ partial eta squared

The same reanalysis strategy was applied to the data of Experiment 4. Six participants reported to have applied both strategies equally frequently for less than half of the blocks (see Appendix Table 26). In contrast to Experiment 3, the mean compliance was overall higher, even though one participant indicated few numbers of “both strategies equally frequently”-blocks. Interestingly, Participant 11, who was eliminated because of low compliance in the primary analysis of Experiment 4 (2.5 SDs below the mean compliance in this experiment), reported only two blocks, with both strategies applied equally frequently. Removing the data of Participant 3 (in addition to Participant 11) resulted in an even larger effect size for the interaction of Target Position × Previous Instruction *F*(2, 34) = 4.070, *p* = 0.026, *ηp*^*2*^ = 0.193 as well as for the planned comparison of quadratic trends *F*(1, 17) = 6.610, *p* = 0.020, *ηp*^*2*^ = 0.280. The Bayesian analysis for the interaction of Target Position × Previous Instruction and corresponding quadratic trends produced moderate evidence (BF_incl_ = 5.010 and BF_incl_ = 5.023, respectively) in favor of the interaction effect.

The elimination of Participants 15 and 04 data from the analysis increased the effect sizes for the interaction of Target Position and Instruction *F*(2, 30) = 4.015, *p* = 0.029, *ηp*^*2*^ = 0.211, planned comparison of quadratic trends *F*(1, 15) = 6.435, *p* = 0.023, *ηp*^*2*^ = 0.300). This Bayesian analysis for the interaction of Target Position × Previous Instruction and corresponding quadratic trends produced moderate evidence (BF_incl_ = 5.232 and BF_incl_ = 4.938, respectively, the interaction effect and quadratic trend) in favor of the interaction effect.

Finally, eliminating the data of participants with very high compliance but fewer blocks of similar strategy application (Participants 08 and 18 with respectively two and zero blocks of equally frequently application of the two strategies) again increased the effect sizes (interaction of Target Position × Previous Instruction *F*(2,26) = 4.374, *p* = 0.023, *ηp*^*2*^ = 0.252, planned comparison of quadratic trends *F*(1, 13) = 10.532, *p* = 0.006, *ηp*^*2*^ = 0.448). This Bayesian analysis for the interaction of Previous Instruction × Target Position and corresponding quadratic trends produced strong (BF_incl_ = 16.393) and very strong (BF_incl_ = 50.077) evidence, respectively, in favor of the interaction effect.

Contrary to the analysis for Experiment 3, the effect sizes and Bayesian inclusion factors for the interaction of Target Position × Previous Instruction and the corresponding quadratic trends improved continuously with each exclusion of participant(s) suspected “bad data”. Especially with the last two participants, who reported high compliance; however, only a few blocks performed both instructions equally frequently. Thus, the second question added valuable information for the decision, which data might be of non-adhering participants in Experiment 4.

A summary of these analyses is presented in Appendix Table 27.

**Table 27:** Summary of the re-analysis of the interaction of interest (Target Position × Previous Instruction) with the corresponding planned comparison of quadratic trends of Experiment 4. Presented are the effect sizes (ηp^2^) of the effects (for full results see the text of Appendix 3). The rows indicate the participants whose data were eliminated from the analysis (successively more from top to bottom), as well as the criteria for the data-elimination; the self-assessed compliance and strategy values

Participants’ data excluded in this step	*N*	ηp^2^ of Target Position × Previous Instruction *(ANOVA/, Planned comparison of quadratic trends)*	BF_incl_ *(ANOVA/, Planned comparison of quadratic trends)*	Mean Compliance over six blocks of Participant	Blocks of applying both strategies
None	20	(*ηp*^*2*^ = 0.135/*ηp*^*2*^ = 0.138)	(2.406/1.343)	*Sample Mean* = 3.63	*Sample Mean* = 3.05
Wo 11	19	(*ηp*^*2*^ = 0.160/*ηp*^*2*^ = 0.255)	(3.132/4.599)	3.69	2
Wo11				2.50	2
and 03	18	(*ηp*^*2*^ = 0.193/*ηp*^*2*^ = 0.280)	(5.010/5.023)	3.00	2
Wo15				3.33	0
and 04	16	(*ηp*^*2*^ = 0.211/*ηp*^*2*^ = 0.300)	(5.232/4.938)	3.33	2
Wo 08				3.67	2
and 18	14	(*ηp*^*2*^ = 0.252/*ηp*^*2*^ = 0.448)	(16.393/50.077)	4.00	0

*wo* without, *N* sample size, *ηp*^*2*^ partial eta squared

In summary, the successive elimination of participants resulted in larger effect sizes for the interaction of interest. This result is consistent with the notion that these participants failed to establish the required attentional set dictated by the instructions (i.e., a more focused or more diffuse allocation of visual attention). The Bayesian analysis for the interaction of interest and the corresponding quadratic trends of the search time results of Experiment 3 supported this notion, especially for Experiment 4, as the Bayes Factors for the quadratic trends were relatively stable at a high level.

This reanalysis aims to demonstrate the value of self-evaluation in assessing participants'adherence to instructions when adherence cannot be controlled through error values or other objective measurements. The results supported the value of self-assessed compliance and, in addition, of an assessment of blocks in which participants reported application of both strategies in comparable frequency for identifying data in which the instructions were not sufficiently adhered to. Combining these sources of information might strengthen the evaluation, as some participants reported a high number of blocks in which they applied the strategies equally frequently, while simultaneously indicating low overall compliance (e.g., Participant 06 in Experiment 3). Several participants, however, reported high adherence to instructions while also indicating that they consistently preferred one strategy in most trial blocks. Especially in Experiment 4, the exclusion of participants only based on a low reported number of blocks in which they followed the instructions equally frequently resulted in a larger effect of instruction-dependent adoption of attentional sets. The self-assessment could be improved by incorporating a third question assessing the relative frequencies after participants indicated a preference for one strategy in a block. In Experiment 3 the last two excluded participants might have followed the instructions, as indicated by compliance values, and the low number of blocks performing the instructions equally frequently might have been misleading.

In conclusion, the analyses presented in this appendix provide tentative support for the notion that effect sizes of voluntary (or facultative) attentional adoption might be compromised by individual participants displaying only low levels of adherence (results of Experiment 4), highlighting the need for an effective method to control for non-adhering participants. Further investigation into the nature of voluntary yet mandatory (or facultative) instructional switches could provide additional insights into endogenous attentional adjustment.

## Footnotes

1 In this article, we use the term “intention-based changes of the attentional set” to refer to changes in the attentional set which occur as a consequence of an intention to do so and which can occur in the absence of any other change in the stimulus environment. Different terms, such as “endogenous” or “top-down”, appear to be used frequently as synonyms. We use the terms “attentional set” and “attentional strategy” interchangeably.

2 Because in that study, arrow cues were used that pointed in the direction of the to-be-attended location, it seems possible that the effect reflects, at least in part, a previously acquired tendency of (non-intentional) orienting in the indicated direction, however. Moreover, participants were told that cue validity was “fairly low” rather than that it was at chance level.

3 In this article, we use the terms *probe task* and *visual search task* interchangeably.

4 Exploratory analysis excluding the ten participants of Experiment 2 who indicated non-adherence to instructions indicated an improvement in the effect size from the diagnostic interaction of Instruction × Target Position *F*(1.73, 15.59) = 1.88, *ηp*^*2*^ = 0.173, *p* = 0.189 and the planned comparison of quadratic trends *F*(1, 9) = 5.19, *ηp*^*2*^ = 0.366, *p* = 0.049, now suggesting a large effect size. The Bayesian analysis for the possible effect of Instruction × Target Position and for corresponding quadratic trends produced anecdotal evidence (BF_excl_ = 1.803) against the interaction, however anecdotal evidence (BF_incl_ = 1.897, respectively) in favor of comparison of quadratic trends of the interaction.
